# Antibodies to *Burkholderia pseudomallei* Outer Membrane Proteins Coupled to Nanovaccines Exhibit Cross-Reactivity to *B. cepacia* Complex and *Pseudomonas aeruginosa* Homologues

**DOI:** 10.3390/microorganisms14010221

**Published:** 2026-01-17

**Authors:** Alexander J. Badten, Susana Oaxaca-Torres, Alfredo G. Torres

**Affiliations:** 1 Department of Microbiology & Immunology, University of Texas Medical Branch, Galveston, TX 77555, USA; 2 Institute for Translational Sciences, University of Texas Medical Branch, Galveston, TX 77555, USA; 3 Office of Faculty Affairs and Development, Meharry Medical College, Nashville, TN 37208, USA

**Keywords:** *Burkholderia pseudomallei*, melioidosis, *Burkholderia cepacia* complex, *Pseudomonas aeruginosa*, vaccines, cross-reactivity, antibodies, T cells

## Abstract

*Burkholderia pseudomallei* complex and *B. cepacia* complex are two evolutionary distinct clades of pathogens causing human disease. Most vaccine efforts have focused on the former group largely due to their biothreat status and global disease burden. It has been proposed that a vaccine could be developed that simultaneously protects against both groups of *Burkholderia* by specifically targeting conserved antigens. Only a few studies have set out to identify which antigens may be optimal targets for such a vaccine. We have previously assessed the ability of three highly conserved *B. pseudomallei* antigens, namely OmpA1, OmpA2, and Pal, coupled to gold nanoparticle vaccines, to protect mice against a homotypic *B. pseudomallei* challenge. Here, we have expanded our study by demonstrating that antibodies to each of these proteins show varying levels of reactivity to homologues in *B. cepacia* complex, with OmpA2 antibodies exhibiting the highest cross-reactivity. Remarkably, some nanovaccine immunized mice, particularly those that received OmpA2, produced antibodies that bind *Pseudomonas aeruginosa*, which harbors distantly related homologous proteins. T cells elicited to Pal and OmpA2 responded to stimulation with *B. cepacia* complex-derived homologues. Our study supports incorporation of these antigens, particularly OmpA2, for the development of a pan-*Burkholderia* vaccine.

## 1. Introduction

The *Bukholderia* genus consists of obligately aerobic, rod-shaped, Gram-negative bacteria which can be found near-ubiquitously across the globe in soil, water-based environments, and colonizing the plant rhizosphere [1,2]. Many *Burkholderia* species benefit humanity through their contribution to nutrient cycling in the soil of crops and by catabolizing manmade environmental pollutants [1,2,3]. However, within this diverse genus, two clades exist that are associated with human disease: the *B. pseudomallei* complex and the *B. cepacia* complex.

Within the *B. pseudomallei* complex, two species are predominantly responsible for cases of human disease: *B. mallei* (*Bm*) and *B. pseudomallei* (*Bpm*). While these pathogens exhibit a high degree of genomic conservation, shared virulence mechanisms, and similar disease manifestations, *Bpm* contributes far more to the total global human disease burden [4,5,6,7]. Melioidosis, the disease caused by *Bpm*, most commonly presents with pneumonia-like symptoms often coinciding with bacteremia [4,5]. This acute, respiratory form of disease is associated with staggeringly high mortality rates ranging from 10% in regions where medical interventions are readily available to upwards of 40% in areas without ready access to effective diagnostics and antibiotic treatment [4,5,8,9]. *Bpm* is found in tropical and subtropical climates, with hotspots of human disease in Northern Australia and Southeast Asia [4,5,6]. However, it has become increasingly evident in recent years that melioidosis cases are significantly underdiagnosed in South and Central America, the Middle East, and Western Sub-Saharan Africa [4,5,6]. Furthermore, global climate change has sparked concerns of increasing disease incidence and spread of *Bpm* to new regions [10,11], which are fears that have been potentially validated by recent reports of autochthonous melioidosis cases in the U.S.A. [12,13] and in Southern Queensland, Australia [14], areas previously not known to harbor the bacteria.

The *B. cepacia* complex consists of over 20 closely related species that are found in a wide range of climates and environments [15,16]. The *B. cepacia* complex species are highly resilient to stressors including nutrient deprivation and biocides, making them relatively common bacterial contaminants of pharmaceuticals and hospital equipment [15]. As a result, *B. cepacia* complex species are common culprits of product recalls and nosocomial outbreaks [17,18]. While these species are known to occasionally infect individuals without any apparent underlying conditions, they are generally regarded as opportunistic pathogens primarily affecting those with cystic fibrosis, chronic granulomatous disease, or weakened immunity [15]. Disease manifestations can vary based on the underlying patient condition, route of transmission, and species- or strain-specific properties of the infecting organism, though pulmonary involvement is relatively common [15,19]. While many of these 20+ species have been isolated from clinical cases, *B. multivorans* (*Bmv*) and *B. cenocepacia* (*Bcc*) are two of the biggest contributors to incidence and mortality, particularly in cystic fibrosis patients [20,21].

While vaccines to *Bpm* and *Bm* have been widely developed on account of their status as potential biothreat agents [5,22,23,24], there are only a small number of publications on *B. cepacia* complex vaccines [25,26,27,28], which can likely be attributed to the smaller population who could benefit from a prophylactic vaccine, the scarcity of animal models that recapitulate human disease, and the inherent complexity of designing a vaccine to >20 species [29]. Interestingly, we and others have previously reported that antibodies and T cells elicited to specific antigens expressed by one species of *Burkholderia* can cross-react to or even protect against other *Burkholderia* species, highlighting the possibility of developing a pan-*Burkholderia* vaccine consisting of highly conserved *Burkholderia* antigens [30,31,32,33,34,35]. However, only a few such studies have looked at inter-clade cross-reactivity of *B. pseudomallei* complex-specific immune responses to *B. cepacia* complex or vice versa [32,34,35], leaving it an open question as to which antigens can be effective in such vaccine. Curiously, one group reported as far back as 1995 that serum antibodies derived from *Pseudomonas aeruginosa* (*Pa*) colonized cystic fibrosis patients can cross-react with *B. cepacia* complex outer membrane proteins [36], though only recently have researchers started to delineate the key cross-reactive antigens, with one group generating a monoclonal antibody capable of reacting to flagellin of both *Bpm* and *Pa* [37] and another that generated polyclonal antibodies to a *Bcc* protein, BCAL2645, capable of functionally cross-reacting to *Pa* [33].

We aim to build upon this initial work by examining three such highly conserved *Bpm* outer membrane proteins that we have previously identified and expressed: BPSL0999 (BpmOmpA1), BPSL2522 (BpmOmpA2), and BPSL2765 (BpmPal) [34]. While we were previously only able to validate the protective properties of nanovaccines containing BpmOmpA1 and BpmOmpA2 in an intranasal C57BL/6 mouse model of melioidosis [34], others have characterized BpmPal as a protective antigen in the BALB/c mouse model [38,39,40], thus warranting further investigation of all three proteins. Herein, we have recombinantly expressed the closest homologues of these proteins from *Bcc* and *Pa* to measure the extent of antibody and T cell cross-reactivity using ELISAs and ex vivo splenocyte antigen recall assays, respectively. We further validated the ability of antibodies to respond to the natively expressed homologous proteins on the surface of a range of bacteria, including *Bpm*, *Bm*, *Bmv*, *Bcc*, and *Pa*. The identification of such broadly conserved surface proteins has far-reaching implications ranging from pan-*Burkholderia* vaccine design to the identification of new broadly conserved therapeutic targets.

## 2. Materials and Methods

### 2.1. In Silico Methodology

Protein sequence FASTA files were obtained from UniProt from the following reference strains: *Bpm* strain K96243, *Bm* strain ATCC 23344, *Bmv* strain ATCC 17616, *Bcc* strain J2315, and *Pa* strain PA01. Homologues of BpmOmpA1, BpmOmpA2, and BpmPal were identified by inputting the associated *Bpm*-derived FASTA file into NCBI’s BLASTp v2.16.0 webtool using the Refseq Select protein database with default blastp parameters (expected threshold of 0.05, word size of 5, BLOSUM62 matrix) [41,42]. For multiple sequence alignment analysis, each of the 15 protein FASTA files were input into Clustal Omega v2.10.0 [43]. The resulting alignment file was visualized with Jalview v2.11.5.0 and residues were colored according to their BLOSUM62 alignment score [44].

### 2.2. Bacterial Strains and Growth Conditions

Genes encoding the proteins of interest were cloned and transformed into *Escherichia coli* BL21(DE3) (New England Biolabs, Ipswich, MA, USA) or Rosetta 2(DE3) pLysS (MilliporeSigma, Burlington, MA, USA) for recombinant protein production. Transformed *E. coli* strains were maintained in Luria–Bertani (LB) medium supplemented with 50 µg/mL kanamycin (Sigma-Aldrich, St. Louis, MO, USA) and, for Rosetta 2(DE3) pLysS, with 34 µg/mL chloramphenicol (Sigma-Aldrich). The attenuated strains *Bpm* Bp82 [45] and *Bm* CLH001 [46], and the wild type strains *Bmv* ATCC 17616, *Bcc* K56-2, and *Pa* PA103 were used for all whole bacteria ELISAs. *Bpm* was propagated in LB supplemented with 100 µg/mL adenine (Sigma-Aldrich) and 5 µg/mL thiamine hydrochloride (Sigma-Aldrich). *Bm* was propagated in LB supplemented with 4% glycerol (Thermo Fisher Scientific) and 200 μM FeSO_4_ (Sigma-Aldrich). *Bmv*, *Bcc*, and *Pa* were cultured in LB without additional supplementation. *E. coli* plates were cultured for 18–24 h at 37 °C prior to its use, while *Burkholderia* and *Pa* plates were cultured for 36–48 h.

### 2.3. Cloning and Recombinant Protein Expression

The cloning, expression, and purification of the *Bpm* proteins was previously reported [34]. The BccPal gene lacking the signal peptide encoding region was PCR amplified from *Bcc* train K56-2 genomic DNA (Ref Seq Accession AM747720). Due to technical challenges arising from the high GC content of *Burkholderia* and *Pa* genomic DNA, *E. coli* codon-optimized gBlocks were instead used for the BccOmpA1, BccOmpA2, PaOmpA1, PaOmpA2, and PaPal genes (Integrated DNA Technologies, Coralville, IA, USA). To mimic the cloning of the *Bpm* proteins [34], the gBlocks encoding BccOmpA1, PaOmpA1, and PaPal did not include the signal peptide encoding sequence while BccOmpA2 and PaOmpA2 gBlocks lacked most of the N-terminal domain. The amplified BccPal gene, gBlocks, and purified pET-30a(+) plasmid (Addgene plasmid # 85761) were separately digested with *Nde*I (New England Biolabs) and *Xho*I (New England Biolabs) overnight at 37 °C before inactivating the enzymes at 65 °C for 20 min using an S1000 Thermal Cycler (Bio-Rad, Hercules, CA, USA). Antarctic phosphatase (New England Biolabs) was added to the linearized plasmid at 37 °C for 30 min and then inactivated at 80 °C for 2 min. Digested plasmid and amplified gene/gBlocks were ran on 0.7% and 1.5% agarose gels, respectively, and the appropriate bands were excised from the gel and processed using a QIAquick Gel Extraction kit (Qiagen, Hilden, Germany) according to manufacturer instructions. Digested amplified gene/gBlocks and linearized plasmid were then mixed at a molar ratio of 3:1 and were ligated with T4 ligase (New England Biolabs) overnight at 16 °C. Five µL of the resulting assembled plasmid was transformed into BL21(DE3) for BccOmpA1 and BccOmpA1 or Rosetta 2(DE3) pLysS for BccOmpA2 and the *Pa* gene-encoding plasmids. Gibson Assembly primers and codon optimized gBlock sequences for the *Bcc* and *Pa* genes are reported in Table S1.

For protein induction, single colonies were transferred to 20 mL LB with appropriate antibiotic selection. Cultures were incubated for 12–18 h at 37 °C with constant shaking at 200 rpm. Cultures were then diluted 1:100 in 1–2 L of fresh, antibiotic-supplemented LB and were grown to an OD_600_ of 0.6, approximately 3–5 h. Protein expression was induced by addition of isopropyl β-D-1-thiogalactopyranoside (GoldBio, St. Louis, MO, USA) at a final concentration of 1 mM. After 4 h of additional culture, the bacteria were centrifuged at 4000× *g* for 10 min at 4 °C, supernatant was discarded, and pellets were stored at −80 °C.

### 2.4. Recombinant Protein Purification

*E. coli* pellets were reconstituted in lysis buffer [50 mM Tris hydrochloride (MilliporeSigma), 500 mM sodium chloride (Thermo Fisher Scientific, Waltham, MA, USA), 20 mM imidazole (Sigma-Aldrich), 10% (*v*/*v*) glycerol (Thermo Fisher Scientific), 1% (*v*/*v*) Triton X-100 (Sigma-Aldrich), 1X cOmplete Protease Inhibitor Cocktail (Roche Life Science), 1X DNase I (Sigma-Aldrich), pH 7.5] on ice with a magnetic stir bar. Lysing of the bacteria was further facilitated by a probe sonicator. The resulting lysate was centrifuged at 22,000× *g* for 1 h at 4 °C and the supernatant was collected and filtered through a 0.45 µm PES membrane. Filtrate was then applied to a HisTrap HP column (Cytiva, Marlborough, MA, USA) connected to an ÄKTA pure liquid chromatography system (Cytiva) using a running buffer consisting of 50 mM Tris hydrochloride, 500 mM sodium chloride, 20 mM imidazole, 10% (*v*/*v*) glycerol, pH 7.5. The column-bound recombinant protein was then eluted by slowly increasing the imidazole concentration to 500 mM and collecting 5 mL fractions. Fractions predominantly consisting of the protein of interest, as ascertained by SDS-PAGE with Coomassie staining, were combined and dialyzed overnight into Dulbecco’s phosphate-buffered saline (PBS; Gibco) at 4 °C. The protein was then concentrated to ≥1.25 mg/mL and applied to a Pierce High-Capacity Endotoxin Removal Column (Thermo Fisher Scientific) for 1 h at room temperature to reduce *E. coli* endotoxin levels. Protein was then diluted to 1 mg/mL in PBS to a final glycerol concentration of 10% (*v*/*v*) and aliquots were flash-frozen in liquid nitrogen before storing at −80 °C.

### 2.5. Gold Nanoparticle Vaccine Synthesis

Proteins were conjugated to ~15 nm spherical gold nanoparticles (AuNP) with a heterobifunctional polyethylene glycol (PEG) linker using our previously established protocol [34]. Unreacted protein in the reaction supernatant was measured via MicroBCA kit (Thermo Fisher Scientific) to back-calculate the amount of conjugated protein. Conjugation efficiencies of the gold nanoparticle vaccines were well within the range reported previously [34].

### 2.6. Mouse Immunizations and Tissue Collection

Six-to-eight-week-old female BALB/c mice were purchased from Charles River Laboratories (Wilmington, MA, USA) and were allowed to acclimate for 1 week prior to experimentation. Animals were housed in microisolator cages under pathogen-free conditions under a 12 h light cycle. Standard rodent chow and water were provided ad libitum.

Mice were immunized intranasally (under light isoflurane sedation) every two weeks for a total of 3 doses with 50 µL (25 µL per nare) of PBS diluted AuNP-conjugated BpmOmpA1 (N = 15), BpmOmpA2 (N = 15), BpmPal (N = 15), PaOmpA1 (N = 10), PaOmpA2 (N = 10), or PaPal (N = 10). Vaccine formulations contained 20 µg/dose VacciGrade CpG ODN 2395 (Invivogen, San Diego, CA, USA) and ~1.5 µg/dose conjugated protein. Saline control animals (N = 10) were administered the same volume of PBS while adjuvant control animals (N = 5) were given an equivalent concentration of PEGylated AuNPs without protein along with 20 µg/dose VacciGrade CpG ODN 2395. Ten days after the final immunization, mice were again sedated and then bled retro-orbitally using microcapillary tubes (Fisher Scientific, Waltham, MA, USA). Microvette tubes containing blood were left at room temperature for 30 min to allow the blood to clot. The tubes were then centrifuged at 2000× *g* for 10 min at 4 °C, and supernatant was collected and stored at −80 °C. Five of the mice immunized with the *Bpm*-derived proteins and the adjuvant control group were humanely euthanized at the same time point and spleens were collected in 5 mL PBS + 2% (*v*/*v*) heat-inactivated fetal bovine serum (FBS; Thermo Fisher Scientific) and kept on ice. A syringe plunger was used to homogenize the spleens on a 70 µm nylon cell strainer, which was subsequently washed with 10 mL PBS + 2% FBS. Cells were centrifuged at 300× *g* for 10 min at 4 °C and supernatant was discarded. Pellets were then resuspended in 5 mL ACK lysing buffer (Thermo Fisher Scientific) for 5 min and then 20 mL PBS was added. Cells were pelleted again in 15 mL complete RPMI 1640 [cRPMI; 10% (*v*/*v*) FBS, 100 units/mL penicillin (Thermo Fisher Scientific), 100 µg/mL streptomycin (Thermo Fisher Scientific), 1X Glutamax (Thermo Fisher Scientific), 1X non-essential amino acids (Thermo Fisher Scientific), 1 mM sodium pyruvate (Thermo Fisher Scientific), and 50 µM cell culture grade β-mercaptoethanol (Sigma-Aldrich)]. An aliquot of splenocytes was stained with trypan blue (Thermo Fisher Scientific) and counted on a hemacytometer to measure viable cell concentration. Cells were centrifuged again into a pellet and resuspended at ≥10^7^ live cells/mL in 90% (*v*/*v*) FBS + 10% (*v*/*v*) cell culture grade dimethyl sulfoxide (Sigma-Aldrich). One mL aliquots in cryotubes were slowly brough to −80 °C overnight and were then transferred to liquid nitrogen for long-term storage.

### 2.7. Serum Enzyme-Linked Immunosorbent Assays (ELISAs)

For recombinant protein ELISAs, the indicated proteins (BpmOmpA1, BpmOmpA2, BpmPal, BccOmpA1, BccOmpA2, BccPal, PaOmpA1, PaOmpA2, PaPal) were coated onto high-binding 96-well plates at 2 µg/mL overnight at 4 °C. Plates were washed four times with PBS + 0.1% (*v*/*v*) Tween 20 (Sigma-Aldrich). Plates were then blocked for 2 h with PBS + 1% (*m*/*v*) bovine serum albumin (Sigma-Aldrich) at room temperature with constant shaking. After another washing step, serial dilutions of vaccination serum were added to the plate for 1.5 h at room temperature. Plates were washed again and then subsequently stained with 1:5000 diluted goat anti-mouse IgG (Southern Biotech, Birmingham, AL, USA) for 1.5 h at room temperature. PBS + 1% BSA was used as the diluent for both the serum and the secondary antibody. After a final wash step, plates were developed with 3,3′,5,5′-tetramethylbenzidine (TMB) substrate (SeraCare, Milford, MA, USA) for 5 min at room temperature and then stopped with 0.18 M sulfuric acid (Sigma-Aldrich). The absorbance was measured at both 450 nm and 650 nm, and A_650_ values were subtracted from A_450_ values to correct for the background absorbance of different wells. Endpoint titers were calculated as the highest dilution at which A_650-450_ values were higher than equivalently diluted saline-immunized mouse serum average signal plus three standard deviations.

Whole bacteria ELISAs were conducted similarly to above, though the Tween 20 concentration of the wash buffer was reduced to 0.025% and the TMB incubation step increased to 30 min. To coat bacteria on the plates, liquid cultures of *Bpm* strain Bp82, *Bm* strain CLH001, *Bmv* strain ATCC 17616, *Bcc* strain K56-2, or *Pa* strain PA103 were grown to stationary phase (12–18 h) at 37 °C with constant shaking at 200 rpm. Bacteria were then centrifuged at 3200× *g* for 10 min and supernatant was discarded. The pellet was then washed once with PBS and then subsequently resuspended to an OD_600_ of 0.4–0.6 in PBS. Finally, 100 µL of bacteria was added to each well of a 96-well high-binding plate, which was allowed to dry overnight in a 37 °C incubator. ELISAs were then carried out as described.

### 2.8. Antigen Recall of Splenocytes

For the ELISpots, 2 × 10^5^ live splenocytes from each mouse (N = 5 per group) were dispensed into separate wells of pre-coated 96-well mouse IFNγ or IL-17A ELISpot plates (R&D Systems, Minneapolis, MN, USA). Cells were stimulated for 24 h at 37 °C with 20 µg/mL of indicated recombinant protein in 100 µL of cRPMI such that splenocytes from the BpmOmpA1 immunization group were stimulated with BpmOmpA1, BccOmpA1, or PaOmpA1, splenocytes from the BpmOmpA2 group were stimulated with BpmOmpA2, BccOmpA2, or PaOmpA2, and splenocytes from the BpmPal group were stimulated with BpmPal, BccPal, or PaPal. As controls, splenocytes from the immunization groups were left for 24 h in cRPMI + 2% (*v*/*v*) PBS or splenocytes from the adjuvant control group were stimulated with each of the individual proteins separately. Plates were then processed according to manufacturer instructions and then imaged on a CTL ImmunoSpot S6 Universal M2 ELISpot Reader (Shaker Heights, OH, USA).

For the flow cytometry experiment, cells from the same mice (N = 5), in technical replicates of 5, were stimulated as described above for 19 h in 96-well tissue culture-treated plates. Negative controls consisted of unstimulated (cRPMI + 2% PBS) splenocytes from the adjuvant control and vaccinated groups. Brefeldin A (Thermo Fisher Scientific) and monensin (Thermo Fisher Scientific) were added to a final concentration of 1X and incubation proceeded for another 5 h. The technical replicates were then combined into a single well of a new 96-well plate. Plates were centrifuged at 300× *g* for 10 min at 4 °C and supernatant was decanted. Cells were washed with PBS, centrifuged, and decanted again. Anti-mouse CD16/32 (BioLegend) was added at a concentration of 5 µg/mL for 10 min at 4 °C. Plates were again centrifuged and decanted, before adding 100 µL/well of surface staining cocktail for 30 min at 4 °C. Surface stain cocktail consisted of experimentally titrated concentrations of anti-mouse CD3e-BUV315 (BD Biosciences, San Jose, CA, USA; clone 145-2C11), anti-mouse CD44-BUV805 (BD Biosciences; clone IM7), anti-mouse CD4-BV510 (BioLegend, San Diego, CA, USA; clone RM4-5), anti-mouse CD69-BV605 (BioLegend; clone H1.2F3), anti-mouse/human B220-BV785 (BioLegend; clone RA3-6B2), anti-mouse CD8a-PerCP/Cy5.5 (Thermo Fisher Scientific; clone 53-6.7), anti-mouse CD25-PE/Fire700 (BioLegend; clone PC61), anti-mouse CD62L-PE/Cy7 (BioLegend; clone MEL-14), and Zombie NIR fixable viability dye (BioLegend) diluted in FACS buffer (BioLegend). Staining controls included unstained cells and fluorescence minus one (FMO) stained cells. The plates were washed again before adding fixation buffer (BioLegend) for 20 min at 4 °C. Plates were washed in 1X permeabilization buffer (BioLegend) and then incubated overnight at 4 °C in intracellular staining cocktail. The intracellular staining cocktail consisted of experimentally titrated concentrations of anti-mouse IL-17A-BV711 (BioLegend; clone TC11-18H10.1), anti-mouse IL-2-BV421 (BioLegend; clone JES6-5H4), anti-mouse IL-4-PE (BioLegend; clone 11B11), and anti-mouse IFNγ-APC (BioLegend; clone XMG1.2) diluted in 1X permeabilization buffer. The next morning, cells were washed in 1X permeabilization buffer and resuspended in FACS buffer immediately prior to application on a BD FACSymphony A5 SE. All data analysis was performed with FlowJo v10.10.

## 3. Results

### 3.1. Identification and Sequence Analysis of OmpA C-like Homologues in Bm, Bmv, Bcc, and Pa

The OmpA family of outer membrane proteins is a group of genetically related, surface-exposed porin proteins that consist of an N-terminal β-barrel porin domain that is embedded in the outer membrane and a C-terminal periplasmic domain that non-covalently interacts with bacterial peptidoglycan [47]. Numerous Gram-negative species express small, globular proteins that bear strong structural similarity to the OmpA C-terminal domain and exhibit peptidoglycan-binding capacity, sometimes referred to as OmpA C-like proteins [34,38,48,49,50,51,52]. While peptidoglycan associated lipoprotein (Pal) is perhaps the most well-studied example [53], other OmpA C-like proteins have also been identified [38,51,52] and evidence exists that this class of proteins can be expressed on the surface of the bacteria where they can be recognized by antibodies [48,49,50,54,55,56]. BpmOmpA1, BpmOmpA2, and BpmPal all belong to this class of OmpA C-like proteins [34,38,49].

We first identified the closest homologues of BpmOmpA1, BpmOmpA2, and BpmPal in *Bm*, *Bmv*, *Bcc*, and *Pa* using protein BLAST v2.16.0 (Table 1) [41,42]. For simplicity, we refer to these homologues as BmOmpA1 (BMA0711), BmOmpA2 (BMA0436), BmPal (BMA2082), BmvOmpA1 (Bmul_0858), BmvOmpA2 (Bmul_2265), BmvPal (Bmul_2588), BccOmpA1 (BCAL2645), BccOmpA2 (BCAL2958), BccPal (BCAL3204), PaOmpA1 (PA0833), PaOmpA2 (PA3692; more commonly referred to as LptF), and PaPal (PA0973; more commonly referred to as OprL). The *Bm* and *Bpm* homologues were all identical at the protein sequence level while *B. cepacia* complex homologues exhibit full or near full (≥99%) sequence coverage and a high degree of sequence identity to their corresponding *Bpm* sequences, ranging from 84.12% to 93.87% (Table 1). Predictably, the *Pa* proteins exhibited markedly lower conservation to their *Bpm* counterparts due to a lack of significant sequence similarity in their N-terminal regions and lower overall sequence identity of the C-terminal domains at 53.08% for PaOmpA1, 39.32% for PaOmpA2, and 46.67% for PaPal (Table 1).

**Figure 1:**
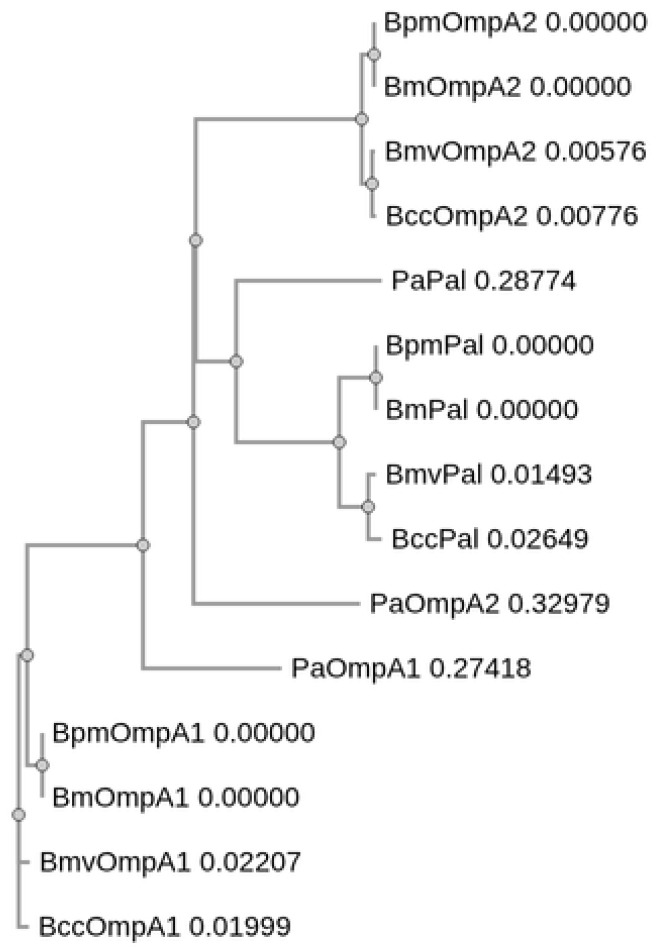
Phylogram of *Burkholderia* and *Pa* OmpA C-like protein amino acid sequences. Created with Clustal Omega v2.10.0 [43].

Given that each of these proteins predominantly consist of the same OmpA C-like domain [34,38], there is a high probability that they arose from a common ancestor. As such, we opted to perform multiple sequence alignment of all 15 protein sequences using Clustal Omega v2.10.0 (Figure 1 and Figure S1) [43]. As expected, the OmpA1 sequences were grouped together, as were the Pal sequences (Figure 1 and Figure S1). While the *Burkholderia* OmpA2 sequences similarly share a clade, PaOmpA2, which notably exhibits the lowest degree of sequence conservation to its closest *Bpm* homolog (Table 1), formed an external branch that shares a common node with the Pal and *Burkholderia* OmpA2 clades (Figure 1). This would suggest that *Pa* lacks a direct homolog of BpmOmpA2, and PaOmpA2 is instead a distinct lineage of the OmpA C-like proteins (Figure 1 and Figure S1). Regardless, because it is the most similar *Pa* protein to BpmOmpA2 and for clarity in the text, we opted to continue referring to the protein as PaOmpA2 herein.

### 3.2. Antibodies Elicited to the Bpm Homologues Exhibit Varying Levels of Cross-Reactivity to Bcc and Pa

**Figure 2:**
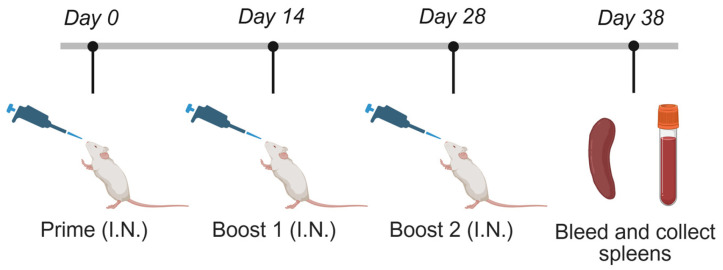
Timeline of mouse immunizations and tissue collection. Created in BioRender. Badten, A. (2026) https://BioRender.com/uxd6kaz.

**Figure 3:**
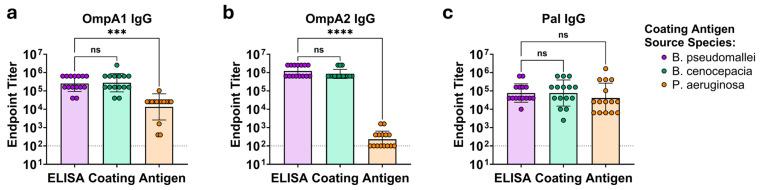
ELISAs using serum from BALB/c mice immunized with (**a**) BpmOmpA1, (**b**) BpmOmpA2, or (**c**) BpmPal. For each vaccination group, three separate ELISAs were performed which differed only in the protein that was coated on the ELISA plates. The BpmOmpA1 group serum was probed against BpmOmpA1, Bcc OmpA1, and PaOmpA1; the BpmOmpA2 group serum was probed against BpmOmpA2, BccOmpA2, and PaOmpA2; and the BpmPal group serum was probed against BpmPal, BccPal, and PaPal. Endpoint titers correspond to the highest dilution at which signal from the immunization serum was >3 SD of the mean signal intensity of the equivalently diluted saline control serum. The limit of detection, represented by the dotted line, was the lowest dilution tested, a factor of 100. Graphs depict geometric mean ± geometric SD. Each ELISA was performed in duplicate, and the graphs depict one representative replicate. Endpoint titers were compared via Friedman test with Dunn’s post hoc tests. Non-significant (ns) *p* ≥ 0.05, (***) *p* < 0.001, (****) *p* < 0.0001.

To assess cross-reactive immunity elicited by these related proteins, we recombinantly expressed BpmOmpA1, BpmOmpA2, BpmPal, BccOmpA1, BccOmpA2, BccPal, PaOmpA1, PaOmpA2, and PaPal (Figure S2). BALB/c mice were immunized with the *Bpm*-derived proteins conjugated to an immunogenic gold nanoparticle (nanovaccine) delivery system that we have extensively characterized (Figure 2) [31,34,57]. We opted to use BALB/c mice for this study instead of the previously utilized C57BL/6 strain because C57BL/6 mice exhibited a relatively poor immune response to BpmPal [34], in contrast to previous reports of its immunogenicity in BALB/c [38,40]. As predicted, immunization of BALB/c mice resulted in more comparable antibody titers to the BpmOmpA1 and BpmOmpA2 vaccines, though BpmPal titers were still slightly lower (Figure 3c, Table S2).

Furthermore, antibodies elicited to all three *Bpm* proteins exhibited indistinguishable reactivity to their *Bcc* homologues, indicating a high degree of cross-reactivity (Figure 3, Table S2). In comparison, reactivity to PaOmpA1 and PaOmpA2 was markedly lower, with 18.9- (*p* = 0.0001) and 5320-fold (*p* < 0.0001) reductions in the reported endpoint titers, respectively (Figure 3a,b, Table S2). The degree of reduced antibody reactivity to these *Pa* homologues is consistent with their relative degree of sequence conservation (Figure 1 and Figure S1 and Table 1). However, despite BpmPal and PaPal having a similar level of sequence conservation as BpmOmpA1 and PaOmpA1, cross-reactivity to the *Pa* homologues was markedly higher for the Pal proteins, with a fold change reduction in endpoint titers of 1.88, a non-statistically significant difference (*p* = 0.6306) (Figure 3c, Table S2). We also probed serum from animals that were immunized with the *Pa*-derived proteins and determined that antibodies to PaOmpA1 and PaPal exhibited consistent but reduced reactivity to the *Bpm*- and *Bcc*-derived homologues (Figure S3). Only 3 of the 10 mice immunized with PaOmpA2 exhibited detectable serum cross-reactivity to the *Bcc*- and *Bpm*-derived homologues (Figure S3), in accordance with PaOmpA2’s lower sequence conservation (Figure 1 and Figure S1 and Table 1).

**Figure 4:**
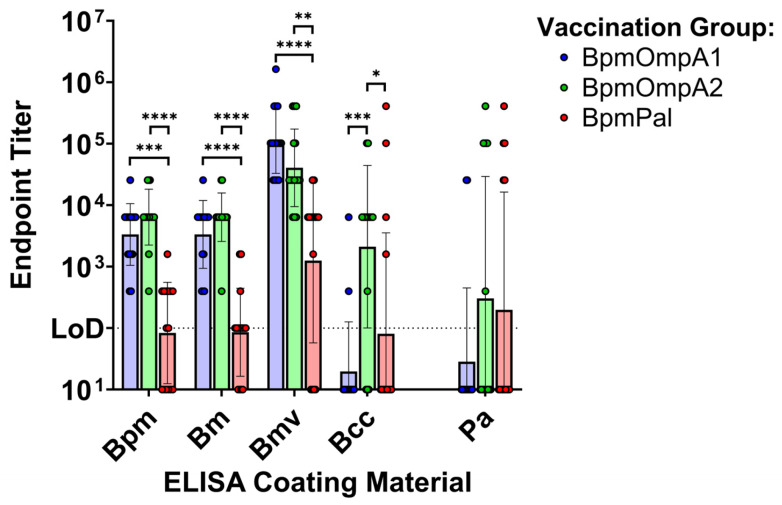
Whole bacteria ELISAs using serum from the immunized BALB/c mice. ELISA plates were coated with intact bacterial cells from *Bpm* strain Bp82, *Bm* strain CLH001, *Bmv* strain ATCC 17616, *Bcc* strain K56-2, or *P. aeruginosa* strain PA103. Endpoint titers correspond to the highest dilution at which signal from the immunization serum was >3 SD of the mean signal intensity of equivalently diluted saline control serum. Graphs depict geometric mean ± geometric SD. The dotted line represents the limit of detection (LoD), the lowest serum dilution tested (1:100). Each ELISA was performed in duplicate, and the graphs depict one representative replicate. Endpoint titers were compared via Kruskal–Wallis with Dunn’s post hoc tests. (*) *p* < 0.05, (**) *p* < 0.01, (***) *p* < 0.001, (****) *p* < 0.0001.

A significant downside of recombinant protein ELISAs is that they do not account for structural differences between the recombinant and native proteins, the native protein’s surface topology, or the presence of other bacterial surface molecules that may physically block the interaction of antibodies with the specific protein. To determine if the serum antibodies elicited by our vaccines can respond to immunologically relevant and exposed epitopes on the surface of the bacteria, we conducted whole bacteria ELISAs (Figure 4, Tables S3 and S4). Serum from the BpmOmpA1 and BpmOmpA2 groups were consistently detected against *Bpm*, *Bm*, and *Bmv*, indicating our vaccination antibodies are consistently capable of recognizing these bacteria (Figure 4, Tables S3 and S4). By comparison, BpmPal serum showed inconsistent reactivity to each of these bacteria, including *Bpm*, indicating that the poorer reactivity has to do with protein-specific properties, such as surface exposure, and not the degree of sequence conservation of the Pal homologues. (Figure 4, Tables S3 and S4). Cross-reactivity of the BpmOmpA1 and BpmPal groups drops markedly against *Bcc* with only a minority of mice exhibiting bacteria-specific reactivity. However, serum from 12 of the 15 BpmOmpA2 animals were still capable of reacting to *Bcc* (Figure 4, Tables S3 and S4). Finally, reactivity to *Pa* was limited, with only a minority of mice in all groups exhibiting any detectable reactivity, in contrast to the recombinant ELISA results (Figure 3 and Figure 4, Tables S3 and S4). However, irrespective of the immunization antigen, 11 of the 13 animals that exhibited serum reactivity to *Pa* also exhibited detectable serum reactivity to each of the *Burkholderia* species, including *Bcc*, which suggests that these mice are responding to one or more specific, highly conserved epitopes (Figure 4, Tables S3 and S4).

### 3.3. BpmOmpA2- and BpmPal-Specific T Cells Cross-React to Their Direct Bcc Homologues

**Figure 5:**
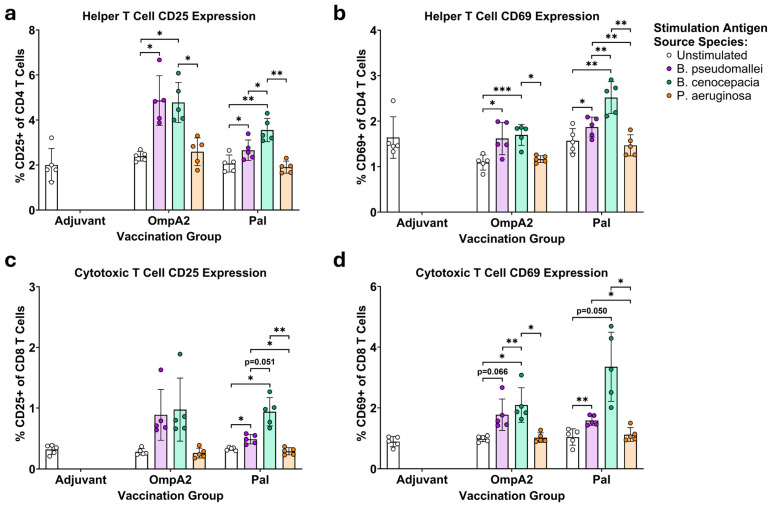
Flow cytometric measurement of the proportion of CD25+ and CD69+ helper T cells (**a**,**b**) and cytotoxic T cells (**c**,**d**) from antigen recalled splenocytes of immunized mice. Graphs depict mean ± SD. Proportions of CD25+ and CD69+ T cells were compared via two-way repeated measures ANOVA with Tukey post hoc tests. (*) *p* < 0.05, (**) *p* < 0.01, (***) *p* < 0.001.

Spleens were collected from the immunized mice and were processed into single-cell suspensions (Figure 2). The recovered splenocytes were then incubated for 24 h with the recombinant protein they were immunized with or its closest homolog in *Bcc* or *Pa*. Cells were then harvested, stained for various lymphocyte markers, and then analyzed on a flow cytometer (Figure 5 and Figures S4 and S5, Table S5). In contrast to our previous study with C57BL/6 mice, the BALB/c animals immunized with BpmOmpA1 did not mount a detectable splenic T cell response to BpmOmpA1, so the degree of T cell cross-reactivity elicited by this antigen could not be assessed [34]. However, splenocytes from BpmPal and BpmOmpA2 immunization groups exhibited a marked increase in the proportion of CD25+ and CD69+ helper and cytotoxic T cells upon stimulation with the *Bpm*-derived antigens, indicating a robust, antigen-specific T cell response (Figure 5, Table S5). In the BpmOmpA2 immunized group, stimulation with homotypic BpmOmpA2 resulted in 2.03- (*p* = 0.0238) and 1.48-fold (*p* = 0.0224) increases in CD25- and CD69-expressing activated helper T cells, respectively, and 3.13- (*p* = 0.1066) and 1.79-fold (*p* = 0.0662) increases in CD25- and CD69-expressing activated cytotoxic T cells, respectively, compared to the unstimulated controls (Figure 5, Table S5). In the BpmPal immunized group, stimulation with the homotypic BpmPal resulted in 1.28- (*p* = 0.0162) and 1.19-fold (*p* = 0.0201) increases in CD25- and CD69-expressing activated helper T cells, respectively, and 1.48- (*p* = 0.0410) and 1.52-fold (*p* = 0.0083) increases in CD25- and CD69-expressing activated cytotoxic T cells, respectively (Figure 5, Table S5).

More importantly, upregulation of these activation markers was also observed in the splenocytes incubated with the *Bcc*-derived homologues, indicating that these T cells cross-react to a significant degree (Figure 5, Table S5). BpmOmpA2 immunized splenocytes that were stimulated with heterologous BccOmpA2 exhibited a highly similar degree of upregulation of activation compared to stimulation with homologous BpmOmpA2. CD25- and CD69-expressing helper T cells increased 2.00- (*p* = 0.0147) and 1.56-fold (*p* = 0.0005), respectively. In contrast, CD25- and CD69-expressing cytotoxic T cells increased 3.44- (*p* = 0.1311) and 2.12-fold (*p* = 0.0310), respectively, compared to unstimulated controls (Figure 5, Table S5). Interestingly, stimulation of the BpmPal immunized splenocytes with BccPal consistently resulted in higher proportions of CD25- and CD69-expressing T cells than stimulation with the homotypic BpmPal (Figure 5, Table S5). Specifically, CD25- and CD69-expressing helper T cells increased 1.72- (*p* = 0.0047) and 1.61-fold (*p* = 0.0043), respectively, while CD25- and CD69-expressing cytotoxic T cells increased 2.83- (*p* = 0.0178) and 3.21-fold (*p* = 0.0500), respectively, compared to the unstimulated controls (Figure 5, Table S5). Stimulation with the *Pa*-derived proteins did not result in detectable levels of T cell activation (Figure 5, Table S5).

**Figure 6:**
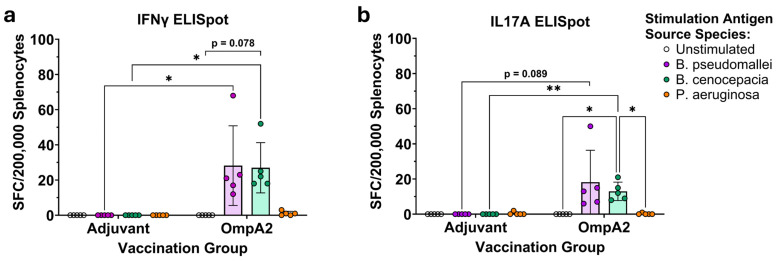
IFNγ (**a**) and IL17A (**b**) ELISpots of antigen recalled splenocytes from the immunized mice. Graphs depict mean ± SD. Spot-forming cells (SFC) of from the different groups were compared via two-way repeated measures ANOVA with Šidák post hoc tests. (*) *p* < 0.05, (**) *p* < 0.01.

To provide confirmatory evidence of this T cell cross-reactivity, we also assayed the stimulated splenocytes via IFNγ and IL-17A ELISpots (Figure 6, Table S6). While splenocytes from the BpmOmpA1 immunization group predictably did not produce meaningful numbers of spot-forming cells (SFC), we were intrigued to find that cells from the BpmPal group also did not produce detectable levels of SFCs to any of the stimulations. Regardless, IFNγ- and IL-17A-producing-cells were detected for the BpmOmpA2 immunization group and followed the same trend as the flow cytometric measurement of activation markers, with comparable levels of SFCs in both the BpmOmpA2 and BccOmpA2 stimulation conditions and minimal SFCs in the negative controls or the PaOmpA2-stimulated group (Figure 5 and Figure 6, Tables S5 and S6). The IL-17A ELISpot results are mirrored in the flow cytometry dataset, with CD4+ T cells in the OmpA2 but not Pal group exhibiting higher staining for intracellular IL-17A when stimulated with the *Bpm*- or *Bcc*-derived recall antigen (Figure S5).

## 4. Discussion

OmpA1, OmpA2, and Pal belong to the same class of OmpA C-like proteins, which are widely expressed across different Gram-negative species [38,48,50,51,52]. Over the years, such proteins have been consistently identified as potential vaccine and therapeutic targets in various bacterial pathogens [51,52,58,59,60], including in *Burkholderia* species [33,34,38,39,40,49,61]. However, few of these studies have directly addressed the relatively high degree of sequence conservation observed in this class of proteins, which may have important implications for their application as vaccine/therapeutic targets. Herein, we have confirmed that antibodies and T cells elicited to these *Bpm*-derived proteins have the capacity to cross-react to more distantly related homologues in *B. cepacia* complex and *Pa* (Figure 3, Figure 4, Figure 5, Figure 6 and Figure S5). The most evident potential benefit is that vaccines and mAbs targeting these proteins may confer broad protection to multiple species, thereby supporting their use as pan-*Burkholderia* antigens as we previously hypothesized [34]. On the other hand, the fact that antibodies could react to much more distantly related *Pa* homologues could indicate that these antibodies may exhibit some reactivity to innocuous bacteria in the environment or as part of the commensal microbiota. For example, the healthy human gut microbiome harbors more closely related bacteria within the *Burkholderiales* order, including the genera *Sutterella*, *Parasutterella*, and *Oxalobacter* [62]. A quick BLASTp v2.17.0 search for the BpmOmpA1, BpmOmpA2, or BpmPal sequences in these genera identifies putative homologues for each with modestly higher sequence conservation than those in *Pa*, indicating that cross-reactivity towards such commensals is a possibility [41,42]. However, given that live-attenuated vaccines, inactivated vaccines, and outer membrane vesicle-derived vaccines [63] also harbor such highly conserved antigens, this may not be of particular concern from a safety standpoint. Indeed, past studies using live-attenuated, inactivated, or conserved subunit antigens have generally found that such immunizations result in only mild or no detectable perturbations to healthy gut commensals [64,65,66]. Regardless, future work will need to carefully assess the pros and cons of targeting such highly conserved proteins.

Regarding cross-reactivity, antibodies elicited towards BpmOmpA1, BpmOmpA2, and BpmPal each exhibited indistinguishable cross-reactivity to their direct, recombinantly expressed *Bcc* homologues (Figure 3, Table S2). By comparison, antibody responses to the natively expressed proteins on the surface of the bacteria were generally more varied (Figure 4, Tables S3 and S4). Given that native proteins may be present on the surface of the bacteria in a consistent orientation, and the fact that other surface proteins and polysaccharides may be physically blocking portions of the native proteins from binding antibodies, we expect that animals that lack serum reactivity to a given species are not producing antibody clones that target conserved and accessible amino acid residues.

Interestingly, only antibodies to BpmOmpA2 showed near-ubiquitous reactivity to all *Burkholderia* species tested, with just 3 of 15 mice showing no reactivity to *Bcc* (Figure 4, Tables S3 and S4). By comparison, antibodies to BpmOmpA1 exhibited full cross-reactivity to *Bm* and *Bmv*, but only 2 of 15 animals showed serum reactivity to *Bcc* (Figure 4, Tables S3 and S4). We noted that BccOmpA1 harbors two unique surface-exposed amino acid substitutions compared to the BpmOmpA1 reference sequence: A174N and N176G (Figure S1). It is possible that this region of the protein is an immunodominant, surface-exposed epitope, and its mutation in Bcc leads to the abrogation of antibody reactivity, though we cannot rule out other factors unique to *Bcc*. Antibodies to BpmPal exhibit inconsistent binding to *Bpm*, indicating it may not be as readily available for binding on the bacteria’s surface or surface-exposed epitopes are not particularly immunodominant, resulting in antibodies predominantly responding to masked epitopes (Figure 4, Tables S3 and S4). Regardless of this, in the mice where *Bpm* reactivity is observed, cross-reactivity followed the same trend as BpmOmpA1, where serum reacts fairly consistently with *Bm* and *Bmv* but sharply drops off when probed against *Bcc* (Figure 4, Tables S3 and S4). While BccPal does harbor a few unique amino acid substitutions compared to BpmPal and BmvPal (Figure S1), none are predicted to be on the surface of the protein, suggesting that other factors may be at play. Regardless, these findings provide strong support for the use of these antigens, particularly BpmOmpA2, as potential pan-*Burkholderia* vaccine targets.

Surprisingly, antibodies to all three *Bpm* proteins also showed a consistent capacity to cross-react to their closest recombinant *Pa* homologues (Figure 3, Table S2). Cross-reactivity to PaOmpA2 was particularly surprising given that it does not appear to be a true direct homolog of BpmOmpA2 (Figure 1) and its relatively low degree of sequence conservation (Table 1, Figure S1). Furthermore, while most mice did not exhibit serum cross-reactivity to *Pa*, a minority of animals in each group that showed broad reactivity to all *Burkholderia* species also exhibited serum cross-reactivity to *Pa* (Figure 4, Tables S3 and S4). One potential explanation for this result is that these mice may be producing antibodies to the most conserved region of the proteins, that being the peptidoglycan-binding domain broadly found in OmpA C-like proteins [67]. However, given that gut microbes also harbor this conserved peptidoglycan-binding motif and are known to induce immune tolerance [68], it may be expected that such regions are poorly immunogenic, which would align with the fact that only a fraction of animals show such broad cross-reactivity (Figure 4, Tables S3 and S4). Future studies will need to conduct epitope mapping experiments to confirm which regions of the proteins are being bound by cross-reactive antibodies. Given that *B. cepacia* complex and *Pa* are both species of particular concern for cystic fibrosis patients [69], targeting both with a single vaccine or therapeutic monoclonal antibody (mAb) merits further exploration.

Because this class of proteins has the ability to induce highly cross-reactive antibodies (Figure 3 and Figure 4) and the evidence that they can be exposed on the surface of many Gram-negative bacterial species (Figure 4) [48,50,54,56,70], it begs the question as to whether mAbs to conserved epitopes on these proteins could be developed for therapeutic application. Indeed, the Tol-Pal system, which is vital for maintaining cell envelope integrity in Gram-negative bacteria, has recently emerged as a promising target for novel antibiotics [53]. While we have thus far been unable to show direct or complement-dependent bactericidal activity using mouse serum antibodies to any of these proteins in the absence of immune cells (unpublished data), Gourlay et al., previously developed a BpmPal-specific mAb capable of opsonizing *Bpm* for subsequent killing by neutrophils via Fc-mediated activity [49]. Given that our current understanding of the Tol-Pal system seems to indicate that Pal predominantly carries out its function while oriented towards the periplasm [53], and it is unclear for what purpose Pal flips its orientation to the outer leaflet of the outer membrane [48,50,54], it is plausible that such Pal-specific antibodies may only mediate protection through Fc- or complement-dependent pathways and not through inhibition of Tol-Pal, though further evaluation will be required to confirm if this is the case. While the function of the OmpA1 and OmpA2 proteins remains to be definitively characterized, Seixas et al., recently showed that polyclonal antibodies to BccOmpA1 are capable of reducing biofilm formation and adherence of *Bcc* to a human cystic fibrosis epithelial cell line, indicating other potential mechanisms of protection that do not solely rely on direct bactericidal/bacteriostatic activity [33,61]. Furthermore, this group showed that such antibodies exhibit similar cross-functionality to *Pa* [33], providing direct evidence that antibodies to this protein could be of value as broadly acting therapeutics.

Outside the scope of vaccine and therapeutic development, these results may also carry important implications for the development of antibody-based diagnostics. In fact, each of these proteins has been studied to varying degrees as diagnostic markers in human melioidosis [71,72,73,74,75,76], horse and human glanders [77,78,79], or *B. cepacia* complex colonization in cystic fibrosis patients [80]. In particular, BpmOmpA2 has consistently been reported as a promising diagnostic antigen for melioidosis and glanders over the years [71,73,74,76,78,79]. Interestingly, while recent work by Settles et al., also concluded that BpmOmpA2 holds strong serodiagnostic potential based on the finding that melioidosis-positive human donors have significantly higher levels of BpmOmpA2-specific antibodies than melioidosis-negative patients, their data also indicates that melioidosis-negative donor samples had relatively high background reactivity to BpmOmpA2 compared to most of the other 46 antigens that were tested [76], which may align with our own finding of interspecies cross-reactive antibodies to this protein (Figure 3 and Figure 4). While the original assessment by Felgner et al., of >1200 *Bpm* antigens as serodiagnostic markers contended that BpmOmpA1 and BpmPal would make for poor antigenic markers based on a high level of background reactivity in healthy donors, which they speculated was due to exposure to other *Burkholderia* species [71], two recent studies have contended that BpmPal has serodiagnostic potential for human melioidosis [75] and *B. cepacia* colonization of cystic fibrosis patients [80]. The former study by Wagner et al., reported that BpmPal is a serodiagnostic marker of melioidosis infection, with pooled melioidosis-positive patient serum exhibiting roughly 24-fold higher signal intensities than pooled healthy control serum; however, the use of pooled serum may have masked any individual control samples with high baseline reactivity [75], which could affect test specificity. Similarly, Peri et al., identified antibodies to BpmPal-derived peptides as potential serodiagnostic markers of *B. cepacia* complex colonization in cystic fibrosis patients, leveraging their sequence conservation between the two *Burkholderia* clades [80]. While the authors ultimately reported a significant difference in reactivity to these peptides in *B. cepacia* complex culture-positive cystic fibrosis patients compared to cystic fibrosis patients colonized with either *Pa* (N = 6), *Achromobacter xylosoxidans* (N = 4), or *Stenotrophomonas maltophilia* (N = 1), their data also indicates that serum from this latter group had sporadic reactivity to the BpmPal peptides, while healthy control samples remained consistently low [80]. Again, this largely aligns with our finds that antibodies elicited to BpmOmpA1, BpmOmpA2, and BpmPal can cross-react to their closest *Pa* homologues (Figure 3 and Figure 4), and we suggest that this sporadic reactivity reported by the authors may be due to cross-reactive antibodies elicited by the patients with *Pa* colonization [80].

Lastly, we also determined that T cells elicited to the BpmPal and BpmOmpA2 are capable of robustly cross-reacting with their *Bcc*-derived homologues (Figure 5, Figure 6 and Figure S5, Tables S5 and S6). Such inter-clade T cell cross-reactivity has scarcely been reported previously, though Musson et al., produced T cell hybridomas to a specific epitope of *Bpm*-derived FliC that cross-reacted to peptides matching FliC sequences in *Bmv*, *Bcc*, and *B. cepacia* [35]. Given that Th1 responses are a known correlate of protection in human melioidosis [81], and Th17 responses are generally regarded as important in mediating protection to respiratory infections such as those caused by *Bpm* and the *B. cepacia* complex [4,15,82], the ability of T cells to cross-react strongly to these evolutionarily distinct *Burkholderia* species adds further support for their use as pan-*Burkholderia* vaccine antigens. Despite antibodies exhibiting consistent cross-reactivity to the recombinant *Pa* proteins (Figure 3, Table S2), we were unable to detect T cell cross-reactivity to them (Figure 5, Figure 6 and Figure S5, Tables S5 and S6). It is possible that specific linear epitopes within the proteins are conserved enough to stimulate cross-reactivity (Figure S1); however, such specific clones of T cells would likely be too rare to be detected using the approaches herein, and instead future work would need to incorporate MHC tetramers into the flow cytometry staining panel or generate T cell hybridomas like those in Musson et al. [35]. On the other hand, while BpmPal-specific antibodies exhibited indistinguishable cross-reactivity to PaPal (Figure 3, Table S2), this protein did not cause detectable levels of T cell activation (Figure 5, Table S5). This may be explained by the differing nature of T cell epitopes, which are always linear, and B cell epitopes, which may be linear or non-contiguous (i.e., “conformational”) [83]. As such, B cells can potentially respond to more varied combinations of surface-exposed amino acids, which may increase the odds of antibody clones being produced that can react to these conserved regions. Additionally, T cell epitopes are limited by what peptides can be presented by the MHC/HLA haplotype of each individual, which may limit the number of potential T cell clones to a greater extent than B cell clones.

In summary, we have demonstrated that the previously described melioidosis nanovaccine antigens BpmOmpA1, BpmOmpA2, and BpmPal can elicit cross-reactive antibodies and T cells to the *B. cepacia* complex, supporting their potential use as pan-*Burkholderia* vaccine antigens. Future work will need to assess whether this cross-reactivity translates to cross-protection.

## Figures and Tables

**Table 1:** BLASTp v2.16.0 comparisons of *Bpm* proteins to closest homologues in *B. mallei* (*Bm*), *B. multivorans* (*Bmv*), *B. cenocepacia* (*Bcc*), and *P. aeruginosa* (*Pa*).

*Bpm* Locus Tag *	*Bm* Sequence Identity (%)	*Bmv* Sequence Identity (%)	*Bcc* Sequence Identity (%)	*Pa* Sequence Coverage (%)	*Pa* Sequence Identity (%)
BPSL0999	100	93.87	93.49	60	53.08
BPSL2522	100	91.52	90.58	52	39.32
BPSL2765	100	86.47	84.12	87	46.67

* BPSL0999 (BpmOmpA1); BPSL2522 (BpmOmpA2); BPSL2765 (BpmPal).

## Data Availability

The original contributions presented in this study are included in the article/Supplementary Material. Further inquiries can be directed to the corresponding author.

## Supplementary Materials

The following supporting information can be downloaded at https://www.mdpi.com/article/10.3390/microorganisms14010221/s1, Figure S1: Multiple sequence alignment of BpmOmpA1 (BPSL0999), BpmOmpA2 (BPSL2522), BpmPal (BPSL2765), BmOmpA1 (BMA0711), BmOmpA2 (BMA0436), BmPal (BMA2082), BmvOmpA1 (Bmul_0858), BmvOmpA2 (Bmul_2265), BmvPal (Bmul_2588), BccOmpA1 (BCAL2645), BccOmpA2 (BCAL2958), BccPal (BCAL3204), PaOmpA1 (PA0833), PaOmpA2 (PA3692), and PaPal (PA0973). Figure S2: SDS-PAGE gel with Coomassie stain of the recombinant proteins used for assessing antibody and T cell cross-reactivity. Figure S3: ELISAs using serum from mice immunized with PaOmpA1, PaOmpA2, or PaPal. Figure S4: Gating strategy employed to analyze the flow cytometry data from the splenocyte antigen recall experiment. Figure S5: Intracellular IL17A fluorescent staining of antigen recalled splenocytes. Table S1: Primers and *E. coli* codon optimized gBlocks used for cloning. Table S2: Corrected *p* values from Friedman tests with Dunn’s post hoc used in Figure 3. Table S3: Whole bacteria ELISA endpoint titers. Table S4: Corrected *p* values from Kruskal–Wallis tests with Dunn’s post hoc used in Figure 4. Table S5: Corrected *p* values from two-way repeated measures ANOVAs with Tukey’s post hoc used in Figure 5. Table S6: Corrected *p* values from two-way repeated measures ANOVAs with Šidák’s post hoc used in Figure 6.

## Abbreviations

The following abbreviations are used in this manuscript:

*Bm*	*Burkholderia mallei*
*Bpm*	*Burkholderia pseudomallei*
*Bmv*	*Burkholderia multivorans*
*Bcc*	*Burkholderia cenocepacia*
*Pa*	*Pseudomonas aeruginosa*
NCBI	National Center for Biotechnology Information
BLASTp	Basic Local Alignment Search Tool (proteins)
BLOSUM	Blocks Substitution Matrix
LB	Luria–Bertani
PCR	Polymerase Chain Reaction
PES	Polyethersulfone
SDS-PAGE	Sodium Dodecyl Sulfate Polyacrylamide Gel Electrophoresis
PBS	Dulbecco’s Phosphate-Buffered Saline
AuNP	Gold Nanoparticle
PEG	Polyethylene glycol
BCA	Bicinchoninic Acid Assay
FBS	Fetal Bovine Serum
cRPMI	Complete Roswell Park Memorial Institute Media
ELISA	Enzyme-Linked Immunosorbent Assays
IgG	Immunoglobulin G
TMB	3,3′,5,5′-tetramethylbenzidine
OD	Optical Density
ELISpot	Enzyme-Linked Immunospot
FACS	Fluorescence-Activated Cell Sorting
ANOVA	Analysis of Variance
SFC	Spot-Forming Cell
mAb	Monoclonal Antibody

## References

[1] Estrada-De Los Santos P., Bustillos-Cristales R., Caballero-Mellado J. (2001). *Burkholderia*, a Genus Rich in Plant-Associated Nitrogen Fixers with Wide Environmental and Geographic Distribution. Appl. Environ. Microbiol..

[2] Eberl L., Vandamme P. (2016). Members of the Genus *Burkholderia*: Good and Bad Guys. F1000Research.

[3] Morya R., Salvachúa D., Thakur I.S. (2020). *Burkholderia*: An Untapped but Promising Bacterial Genus for the Conversion of Aromatic Compounds. Trends Biotechnol..

[4] Wiersinga W.J., Virk H.S., Torres A.G., Currie B.J., Peacock S.J., Dance D.A.B., Limmathurotsakul D. (2018). Melioidosis. Nat. Rev. Dis. Primers.

[5] Meumann E.M., Limmathurotsakul D., Dunachie S.J., Wiersinga W.J., Currie B.J. (2024). *Burkholderia pseudomallei* and Melioidosis. Nat. Rev. Microbiol..

[6] Limmathurotsakul D., Golding N., Dance D.A., Messina J.P., Pigott D.M., Moyes C.L., Rolim D.B., Bertherat E., Day N.P., Peacock S.J. (2016). Predicted Global Distribution of *Burkholderia pseudomallei* and Burden of Melioidosis. Nat. Microbiol..

[7] Van Zandt K.E., Greer M.T., Gelhaus H.C. (2013). Glanders: An Overview of Infection in Humans. Orphanet J. Rare Dis..

[8] Currie B.J. (2015). Melioidosis: Evolving Concepts in Epidemiology, Pathogenesis, and Treatment. Semin. Respir. Crit. Care Med..

[9] Currie B.J., Mayo M., Ward L.M., Kaestli M., Meumann E.M., Webb J.R., Woerle C., Baird R.W., Price R.N., Marshall C.S. (2021). The Darwin Prospective Melioidosis Study: A 30-Year Prospective, Observational Investigation. Lancet Infect. Dis..

[10] Merritt A.J., Inglis T.J.J. (2017). The Role of Climate in the Epidemiology of Melioidosis. Curr. Trop. Med. Rep..

[11] Chai L.Y.A., Fisher D. (2018). Earth, Wind, Rain, and Melioidosis. Lancet Planet. Health.

[12] HAN Archive-00470|Health Alert Network (HAN). https://archive.cdc.gov/www_cdc_gov/han/2022/han00470.html.

[13] Brennan S., Thompson J.M., Gulvik C.A., Paisie T.K., Elrod M.G., Gee J.E., Schrodt C.A., DeBord K.M., Richardson B.T., Drenzek C. (2025). Related Melioidosis Cases with Unknown Exposure Source, Georgia, USA, 1983–2024. Emerg. Infect. Dis..

[14] Gassiep I., Grey V., Thean L.J., Farquhar D., Clark J.E., Ariotti L., Graham R., Jennison A.V., Bergh H., Anuradha S. (2023). Expanding the Geographic Boundaries of Melioidosis in Queensland, Australia. Am. J. Trop. Med. Hyg..

[15] Tavares M., Kozak M., Balola A., Sá-Correia I. (2020). *Burkholderia cepacia* Complex Bacteria: A Feared Contamination Risk in Water-Based Pharmaceutical Products. Clin. Microbiol. Rev..

[16] Velez L.S., Aburjaile F.F., Farias A.R.G., Baia A.D.B., Oliveira W.J., Silva A.M.F., Benko-Iseppon A.M., Azevedo V., Brenig B., Ham J.H. (2023). *Burkholderia semiarida* sp. Nov. and *Burkholderia sola* sp. Nov., Two Novel *B. cepacia* Complex Species Causing Onion Sour Skin. Syst. Appl. Microbiol..

[17] Torbeck L., Raccasi D., Guilfoyle D.E., Friedman R.L., Hussong D. (2011). *Burkholderia cepacia*: This Decision Is Overdue. PDA J. Pharm. Sci. Technol..

[18] Häfliger E., Atkinson A., Marschall J. (2020). Systematic Review of Healthcare-Associated *Burkholderia cepacia* Complex Outbreaks: Presentation, Causes and Outbreak Control. Infect. Prev. Pract..

[19] CDC About *Burkholderia cepacia* Complex. https://www.cdc.gov/b-cepacia/about/index.html.

[20] Scoffone V.C., Chiarelli L.R., Trespidi G., Mentasti M., Riccardi G., Buroni S. (2017). *Burkholderia cenocepacia* Infections in Cystic Fibrosis Patients: Drug Resistance and Therapeutic Approaches. Front. Microbiol..

[21] Zlosnik J.E.A., Henry D.A., Hird T.J., Hickman R., Campbell M., Cabrera A., Laino Chiavegatti G., Chilvers M.A., Sadarangani M. (2020). Epidemiology of *Burkholderia* Infections in People with Cystic Fibrosis in Canada between 2000 and 2017. Ann. ATS.

[22] Badten A.J., Torres A.G. (2024). *Burkholderia pseudomallei* Complex Subunit and Glycoconjugate Vaccines and Their Potential to Elicit Cross-Protection to *Burkholderia cepacia* Complex. Vaccines.

[23] Select Agents and Toxins List|Federal Select Agent Program. https://www.selectagents.gov/sat/list.htm.

[24] Sengyee S., Weiby S.B., Rok I.T., Burtnick M.N., Brett P.J. (2025). Melioidosis Vaccines: Recent Advances and Future Directions. Front. Immunol..

[25] Bertot G.M., Restelli M.A., Galanternik L., Aranibar Urey R.C., Valvano M.A., Grinstein S. (2007). Nasal Immunization with *Burkholderia multivorans* Outer Membrane Proteins and the Mucosal Adjuvant Adamantylamide Dipeptide Confers Efficient Protection against Experimental Lung Infections with *B. multivorans* and *B. cenocepacia*. Infect. Immun..

[26] Makidon P.E., Knowlton J., Groom J.V., Blanco L.P., LiPuma J.J., Bielinska A.U., Baker J.R. (2010). Induction of Immune Response to the 17 kDa OMPA *Burkholderia cenocepacia* Polypeptide and Protection against Pulmonary Infection in Mice after Nasal Vaccination with an OMP Nanoemulsion-Based Vaccine. Med. Microbiol. Immunol..

[27] McClean S., Healy M.E., Collins C., Carberry S., O’Shaughnessy L., Dennehy R., Adams Á., Kennelly H., Corbett J.M., Carty F. (2016). Linocin and OmpW Are Involved in Attachment of the Cystic Fibrosis-Associated Pathogen *Burkholderia cepacia* Complex to Lung Epithelial Cells and Protect Mice against Infection. Infect. Immun..

[28] Gawad W.E., Nagy Y.I., Samir T.M., Mansour A.M.I., Helmy O.M. (2025). Cyclic Di AMP Phosphodiesterase Nanovaccine Elicits Protective Immunity against *Burkholderia cenocepacia* Infection in Mice. Npj Vaccines.

[29] Pradenas G.A., Myers J.N., Torres A.G. (2017). Characterization of the *Burkholderia cenocepacia tonB* Mutant as a Potential Live Attenuated Vaccine. Vaccines.

[30] Rongkard P., Kronsteiner B., Hantrakun V., Jenjaroen K., Sumonwiriya M., Chaichana P., Chumseng S., Chantratita N., Wuthiekanun V., Fletcher H.A. (2020). Human Immune Responses to Melioidosis and Cross-Reactivity to Low-Virulence *Burkholderia* Species, Thailand. Emerg. Infect. Dis..

[31] Tapia D., Sanchez-Villamil J.I., Stevenson H.L., Torres A.G. (2021). Multicomponent Gold-Linked Glycoconjugate Vaccine Elicits Antigen-Specific Humoral and Mixed TH1-TH17 Immunity, Correlated with Increased Protection against *Burkholderia pseudomallei*. mBio.

[32] Burtnick M.N., Dance D.A.B., Vongsouvath M., Newton P.N., Dittrich S., Sendouangphachanh A., Woods K., Davong V., Kenna D.T.D., Saiprom N. (2024). Identification of *Burkholderia cepacia* Strains That Express a *Burkholderia pseudomallei*-like Capsular Polysaccharide. Microbiol. Spectr..

[33] Seixas A.M.M., Gomes S.C., Silva C., Moreira L.M., Leitão J.H., Sousa S.A. (2024). A Polyclonal Antibody against a *Burkholderia cenocepacia* OmpA-like Protein Strongly Impairs *Pseudomonas aeruginosa* and *B. multivorans* Virulence. Vaccines.

[34] Badten A.J., Oaxaca-Torres S., Basu R.S., Gagnon M.G., Torres A.G. (2025). Evaluation of Highly Conserved *Burkholderia pseudomallei* Outer Membrane Proteins as Protective Antigens against Respiratory Melioidosis. Npj Vaccines.

[35] Musson J.A., Reynolds C.J., Rinchai D., Nithichanon A., Khaenam P., Favry E., Spink N., Chu K.K., De Soyza A., Bancroft G.J. (2014). CD4^+^ T Cell Epitopes of FliC Conserved between Strains of *Burkholderia*—Implications for Vaccines against Melioidosis and *cepacia* Complex in Cystic Fibrosis. J. Immunol..

[36] Lacy D.E., Smith A.W., Stableforth D.E., Smith G., Weller P.H., Brown M.R.W. (1995). Serum IgG Response to *Burkholderia cepacia* Outer Membrane Antigens in Cystic Fibrosis: Assessment of Cross-Reactivity with *Pseudomonas aeruginosa*. FEMS Immunol. Med. Microbiol..

[37] Mateu-Borrás M., Dublin S.R., Kang J., Monroe H.L., Sen-Kilic E., Miller S.J., Witt W.T., Chapman J.A., Pyles G.M., Nallar S.C. (2024). Novel Broadly Reactive Monoclonal Antibody Protects against *Pseudomonas aeruginosa* Infection. Infect. Immun..

[38] Hara Y., Mohamed R., Nathan S. (2009). Immunogenic *Burkholderia pseudomallei* Outer Membrane Proteins as Potential Candidate Vaccine Targets. PLoS ONE.

[39] Champion O.L., Gourlay L.J., Scott A.E., Lassaux P., Conejero L., Perletti L., Hemsley C., Prior J., Bancroft G., Bolognesi M. (2016). Immunisation with Proteins Expressed during Chronic Murine Melioidosis Provides Enhanced Protection against Disease. Vaccine.

[40] Dyke J.S., Huertas-Diaz M.C., Michel F., Holladay N.E., Hogan R.J., He B., Lafontaine E.R. (2020). The Peptidoglycan-Associated Lipoprotein Pal Contributes to the Virulence of *Burkholderia mallei* and Provides Protection against Lethal Aerosol Challenge. Virulence.

[41] Altschul S.F., Gish W., Miller W., Myers E.W., Lipman D.J. (1990). Basic Local Alignment Search Tool. J. Mol. Biol..

[42] Camacho C., Coulouris G., Avagyan V., Ma N., Papadopoulos J., Bealer K., Madden T.L. (2009). BLAST+: Architecture and Applications. BMC Bioinform..

[43] Madeira F., Madhusoodanan N., Lee J., Eusebi A., Niewielska A., Tivey A.R.N., Lopez R., Butcher S. (2024). The EMBL-EBI Job Dispatcher Sequence Analysis Tools Framework in 2024. Nucleic Acids Res..

[44] Waterhouse A.M., Procter J.B., Martin D.M.A., Clamp M., Barton G.J. (2009). Jalview Version 2—A Multiple Sequence Alignment Editor and Analysis Workbench. Bioinformatics.

[45] Propst K.L., Mima T., Choi K.-H., Dow S.W., Schweizer H.P. (2010). A *Burkholderia pseudomallei* Δ*purM* Mutant Is Avirulent in Immunocompetent and Immunodeficient Animals: Candidate Strain for Exclusion from Select-Agent Lists. Infect. Immun..

[46] Hatcher C.L., Mott T.M., Muruato L.A., Sbrana E., Torres A.G. (2016). *Burkholderia mallei* CLH001 Attenuated Vaccine Strain Is Immunogenic and Protects against Acute Respiratory Glanders. Infect. Immun..

[47] Confer A.W., Ayalew S. (2013). The OmpA Family of Proteins: Roles in Bacterial Pathogenesis and Immunity. Vet. Microbiol..

[48] Michel L.V., Snyder J., Schmidt R., Milillo J., Grimaldi K., Kalmeta B., Khan M.N., Sharma S., Wright L.K., Pichichero M.E. (2013). Dual Orientation of the Outer Membrane Lipoprotein P6 of Nontypeable *Haemophilus influenzae*. J. Bacteriol..

[49] Gourlay L.J., Peri C., Ferrer-Navarro M., Conchillo-Solé O., Gori A., Rinchai D., Thomas R.J., Champion O.L., Michell S.L., Kewcharoenwong C. (2013). Exploiting the *Burkholderia pseudomallei* Acute Phase Antigen BPSL2765 for Structure-Based Epitope Discovery/Design in Structural Vaccinology. Chem. Biol..

[50] Michel L.V., Shaw J., MacPherson V., Barnard D., Bettinger J., D’Arcy B., Surendran N., Hellman J., Pichichero M.E. (2015). Dual Orientation of the Outer Membrane Lipoprotein Pal in *Escherichia coli*. Microbiology.

[51] Yang F., Gu J., Zou J., Lei L., Jing H., Zhang J., Zeng H., Zou Q., Lv F., Zhang J. (2018). PA0833 Is an OmpA C-Like Protein That Confers Protection Against *Pseudomonas aeruginosa* Infection. Front. Microbiol..

[52] Cheng X., Chen Z., Gao C., Zhang Y., Yang L., Wan J., Wei Y., Zeng S., Zhang Y., Zhang Y. (2023). Structural and Biological Insights into Outer Membrane Protein Lipotoxin F of *Pseudomonas aeruginosa*: Implications for Vaccine Application. Int. J. Biol. Macromol..

[53] Szczepaniak J., Webby M.N. (2024). The Tol Pal System Integrates Maintenance of the Three Layered Cell Envelope. Npj Antimicrob. Resist..

[54] Wilson M.M., Bernstein H.D. (2016). Surface Exposed Lipoproteins: An Emerging Secretion Phenomenon in Gram-Negative Bacteria. Trends Microbiol..

[55] Sousa S.A., Seixas A.M.M., Mandal M., Rodríguez-Ortega M.J., Leitão J.H. (2020). Characterization of the *Burkholderia cenocepacia* J2315 Surface-Exposed Immunoproteome. Vaccines.

[56] Cole G.B., Bateman T.J., Moraes T.F. (2020). The Surface Lipoproteins of Gram-Negative Bacteria: Protectors and Foragers in Harsh Environments. J. Biol. Chem..

[57] Gregory A.E., Judy B.M., Qazi O., Blumentritt C.A., Brown K.A., Shaw A.M., Torres A.G., Titball R.W. (2015). A Gold Nanoparticle-Linked Glycoconjugate Vaccine against *Burkholderia mallei*. Nanomed. Nanotechnol. Biol. Med..

[58] Gao C., Yang F., Wang Y., Liao Y., Zhang J., Zeng H., Zou Q., Gu J. (2017). Vaccination with a Recombinant OprL Fragment Induces a Th17 Response and Confers Serotype-Independent Protection against *Pseudomonas aeruginosa* Infection in Mice. Clin. Immunol..

[59] Chen Y., Yang Z., Dong Y., Chen Y. (2020). Recombinant PAL/PilE/FlaA DNA Vaccine Provides Protective Immunity against *Legionella pneumophila* in BALB/c Mice. BMC Biotechnol..

[60] Solanki V., Tiwari M., Tiwari V. (2023). Investigation of Peptidoglycan-Associated Lipoprotein of *Acinetobacter baumannii* and Its Interaction with Fibronectin to Find Its Therapeutic Potential. Infect. Immun..

[61] Seixas A.M.M., Sousa S.A., Feliciano J.R., Gomes S.C., Ferreira M.R., Moreira L.M., Leitão J.H. (2021). A Polyclonal Antibody Raised against the *Burkholderia cenocepacia* OmpA-like Protein BCAL2645 Impairs the Bacterium Adhesion and Invasion of Human Epithelial Cells In Vitro. Biomedicines.

[62] Species Summary|Microbiome Atlas. https://www.microbiomeatlas.org/species_summary.php.

[63] Baker S.M., Settles E.W., Davitt C., Gellings P., Kikendall N., Hoffmann J., Wang Y., Bitoun J., Lodrigue K.-R., Sahl J.W. (2021). *Burkholderia pseudomallei* OMVs Derived from Infection Mimicking Conditions Elicit Similar Protection to a Live-Attenuated Vaccine. Npj Vaccines.

[64] Eloe-Fadrosh E.A., McArthur M.A., Seekatz A.M., Drabek E.F., Rasko D.A., Sztein M.B., Fraser C.M. (2013). Impact of Oral Typhoid Vaccination on the Human Gut Microbiota and Correlations with *S. typhi*-Specific Immunological Responses. PLoS ONE.

[65] Hays M.P., Ericsson A.C., Yang Y., Hardwidge P.R. (2016). Vaccinating with Conserved *Escherichia coli* Antigens Does Not Alter the Mouse Intestinal Microbiome. BMC Res. Notes.

[66] Vieira de Araujo A.E., Conde L.V., da Silva Junior H.C., de Almeida Machado L., Lara F.A., Chapeaurouge A., Pauer H., Pires Hardoim C.C., Martha Antunes L.C., D’Alincourt Carvalho-Assef A.P. (2021). Cross-Reactivity and Immunotherapeutic Potential of BamA Recombinant Protein from *Acinetobacter baumannii*. Microbes Infect..

[67] Park J.S., Lee W.C., Yeo K.J., Ryu K.-S., Kumarasiri M., Hesek D., Lee M., Mobashery S., Song J.H., Kim S.I. (2012). Mechanism of Anchoring of OmpA Protein to the Cell Wall Peptidoglycan of the Gram-Negative Bacterial Outer Membrane. FASEB J..

[68] Shao T., Hsu R., Rafizadeh D.L., Wang L., Bowlus C.L., Kumar N., Mishra J., Timilsina S., Ridgway W.M., Gershwin M.E. (2023). The Gut Ecosystem and Immune Tolerance. J. Autoimmun..

[69] Erfanimanesh S., Emaneini M., Modaresi M.R., Feizabadi M.M., Halimi S., Beigverdi R., Nikbin V.S., Jabalameli F. (2022). Distribution and Characteristics of Bacteria Isolated from Cystic Fibrosis Patients with Pulmonary Exacerbation. Can. J. Infect. Dis. Med. Microbiol..

[70] Spinola S.M., Griffiths G.E., Bogdan J., Menegus M.A. (1992). Characterization of an 18,000-Molecular-Weight Outer Membrane Protein of *Haemophilus ducreyi* That Contains a Conserved Surface-Exposed Epitope. Infect. Immun..

[71] Felgner P.L., Kayala M.A., Vigil A., Burk C., Nakajima-Sasaki R., Pablo J., Molina D.M., Hirst S., Chew J.S.W., Wang D. (2009). A *Burkholderia pseudomallei* Protein Microarray Reveals Serodiagnostic and Cross-Reactive Antigens. Proc. Natl. Acad. Sci. USA.

[72] Yang Z.R., Lertmemongkolchai G., Tan G., Felgner P.L., Titball R. (2009). A Genetic Programming Approach for *Burkholderia pseudomallei* Diagnostic Pattern Discovery. Bioinformatics.

[73] Hara Y., Chin C.-Y., Mohamed R., Puthucheary S.D., Nathan S. (2013). Multiple-Antigen ELISA for Melioidosis—A Novel Approach to the Improved Serodiagnosis of Melioidosis. BMC Infect. Dis..

[74] Kohler C., Dunachie S.J., Müller E., Kohler A., Jenjaroen K., Teparrukkul P., Baier V., Ehricht R., Steinmetz I. (2016). Rapid and Sensitive Multiplex Detection of *Burkholderia pseudomallei*-Specific Antibodies in Melioidosis Patients Based on a Protein Microarray Approach. PLoS Negl. Trop. Dis..

[75] Wagner G.E., Stanjek T.F.P., Albrecht D., Lipp M., Dunachie S.J., Föderl-Höbenreich E., Riedel K., Kohler A., Steinmetz I., Kohler C. (2023). Deciphering the Human Antibody Response against *Burkholderia pseudomallei* during Melioidosis Using a Comprehensive Immunoproteome Approach. Front. Immunol..

[76] Settles E.W., Sonderegger D., Shannon A.B., Celona K.R., Lederer R., Yi J., Seavey C., Headley K., Mbegbu M., Harvey M. (2023). Development and Evaluation of a Multiplex Serodiagnostic Bead Assay (BurkPx) for Accurate Melioidosis Diagnosis. PLoS Negl. Trop. Dis..

[77] Varga J.J., Vigil A., DeShazer D., Waag D.M., Felgner P., Goldberg J.B. (2012). Distinct Human Antibody Response to the Biological Warfare Agent *Burkholderia mallei*. Virulence.

[78] Wagner G.E., Berner A., Lipp M., Kohler C., Assig K., Lichtenegger S., Saqib M. (2023). Protein Microarray-Guided Development of a Highly Sensitive and Specific Dipstick Assay for Glanders Serodiagnostics. Clin. Microbiol..

[79] Ichikawa Y., Iinuma Y., Okagawa T., Shimbo R., Enkhtuul B., Kjurtsbaatar O., Kinoshita Y., Niwa H., Aoshima K., Kobayashi A. (2025). Comparison of Immunogenicity of 17 *Burkholderia mallei* Antigens and Whole Cell Lysate Using Indirect ELISA. J. Vet. Med. Sci..

[80] Peri C., Gori A., Gagni P., Sola L., Girelli D., Sottotetti S., Cariani L., Chiari M., Cretich M., Colombo G. (2016). Evolving Serodiagnostics by Rationally Designed Peptide Arrays: The *Burkholderia* Paradigm in Cystic Fibrosis. Sci. Rep..

[81] Jenjaroen K., Chumseng S., Sumonwiriya M., Ariyaprasert P., Chantratita N., Sunyakumthorn P., Hongsuwan M., Wuthiekanun V., Fletcher H.A., Teparrukkul P. (2015). T-Cell Responses Are Associated with Survival in Acute Melioidosis Patients. PLoS Negl. Trop. Dis..

[82] Rathore J.S., Wang Y. (2016). Protective Role of Th17 Cells in Pulmonary Infection. Vaccine.

[83] Forsström B., Bisławska Axnäs B., Rockberg J., Danielsson H., Bohlin A., Uhlen M. (2015). Dissecting Antibodies with Regards to Linear and Conformational Epitopes. PLoS ONE.

