# Supplementary material for: Antibodies to Burkholderia pseudomallei Outer Membrane Proteins Coupled to Nanovaccines Exhibit Cross-Reactivity to B. cepacia Complex and Pseudomonas aeruginosa Homologues
Source: Microorganisms. 2026 Jan 17;14(1):221. doi: 10.3390/microorganisms14010221 (PMC12844004; doi:10.3390/microorganisms14010221)
Supplement: Supplementary file 1 [file microorganisms-14-00221-s001.zip › microorganisms-4074656-supplementary.pdf]

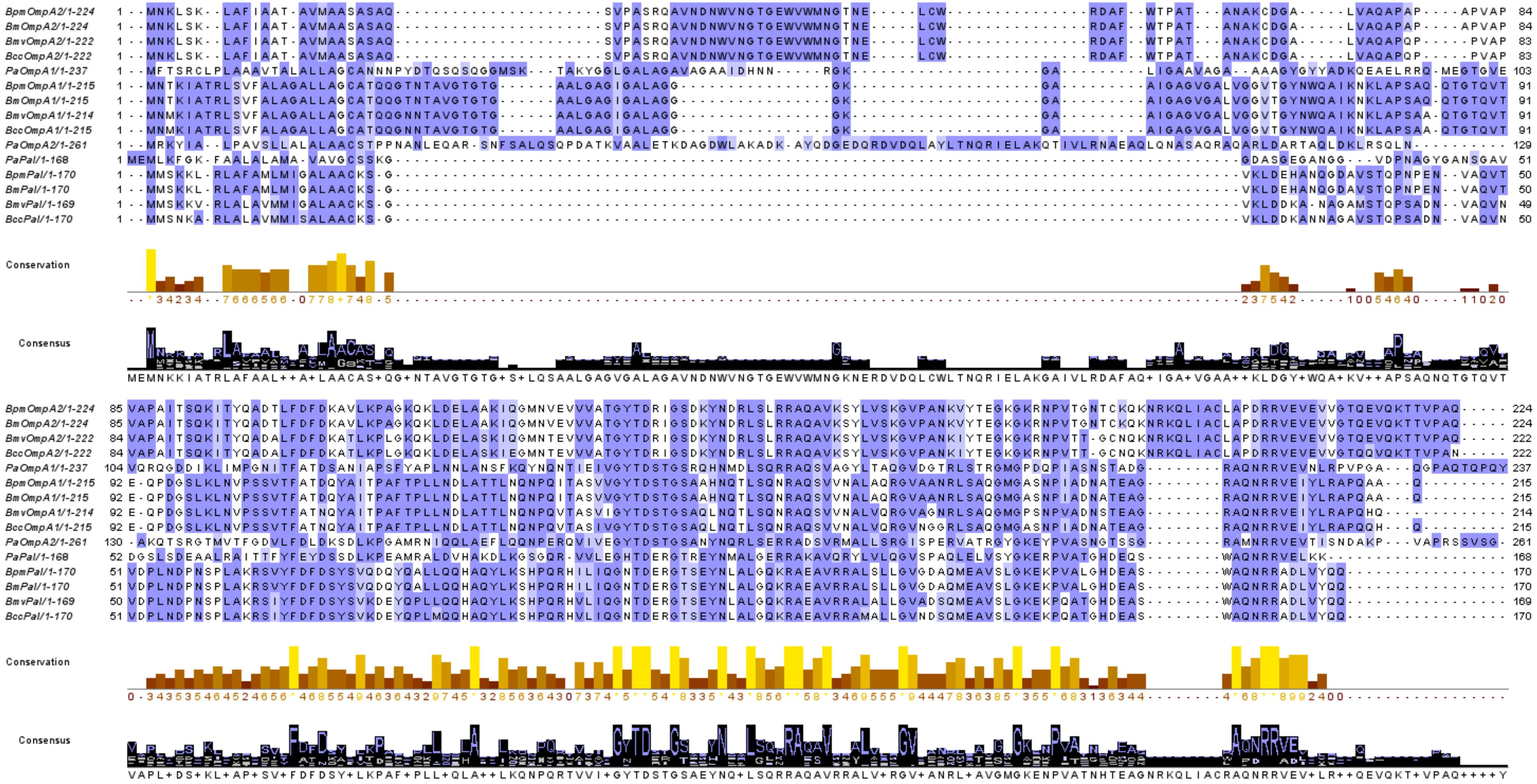

Figure S1. Multiple sequence alignment of BpmOmpA1 (BPSL0999), BpmOmpA2 (BPSL2522), BpmPal (BPSL2765), BmOmpA1 (BMA0711), BmOmpA2 (BMA0436), BmPal (BMA2082), BmvOmpA1 (Bmul_0858), BmvOmpA2 (Bmul_2265), BmvPal (Bmul_2588), BccOmpA1 (BCAL2645), BccOmpA2 (BCAL2958), BccPal (BCAL3204), PaOmpA1 (PA0833), PaOmpA2 (PA3692), and PaPal (PA0973). Created with Clustal Omega and visualized with Jalview v2.11.5.0. Residues are colored according to their BLOSUM62 alignment score.

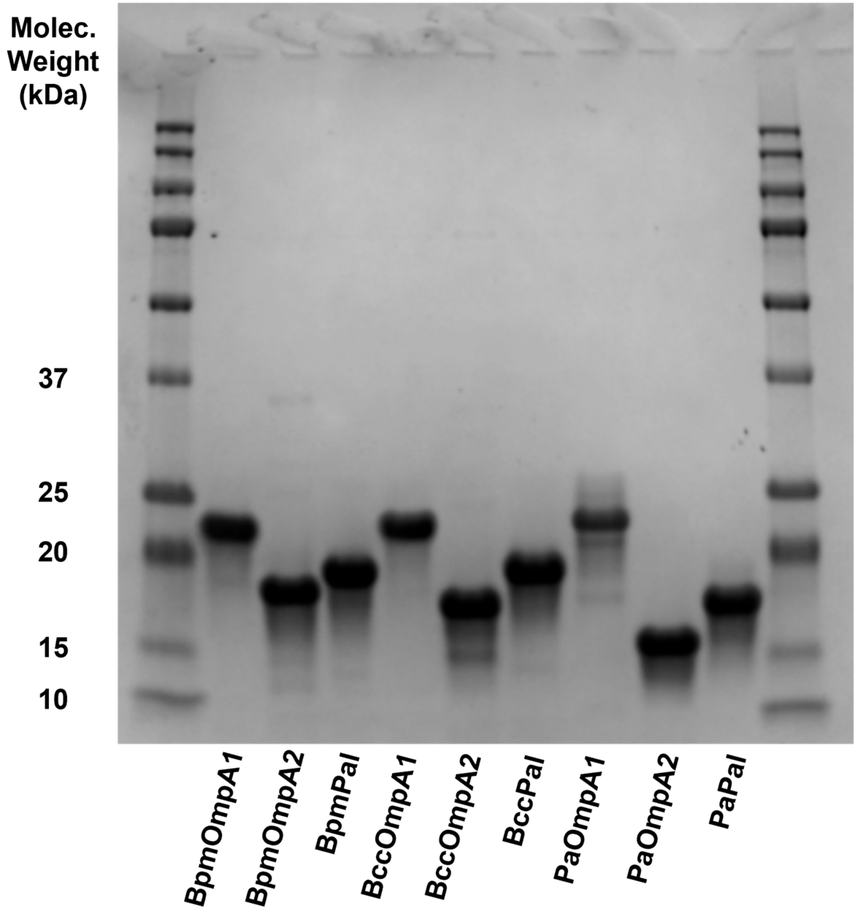

Figure S2: SDS-PAGE gel with Coomassie stain of the recombinant proteins used for assessing antibody and T cell cross-reactivity. Expasy predicted molecular weights: BpmOmpA1 = 20.2 kDa, BpmOmpA2 = 16.3 kDa, BpmPal = 17.5 kDa, BccOmpA1 = 20.3 kDa, BccOmpA2 = 16.2 kDa, BccPal = 17.5 kDa, PaOmpA1 = 23.5 kDa. PaOmpA2 = 15.3 kDa, PaPal = 16.6 kDa.

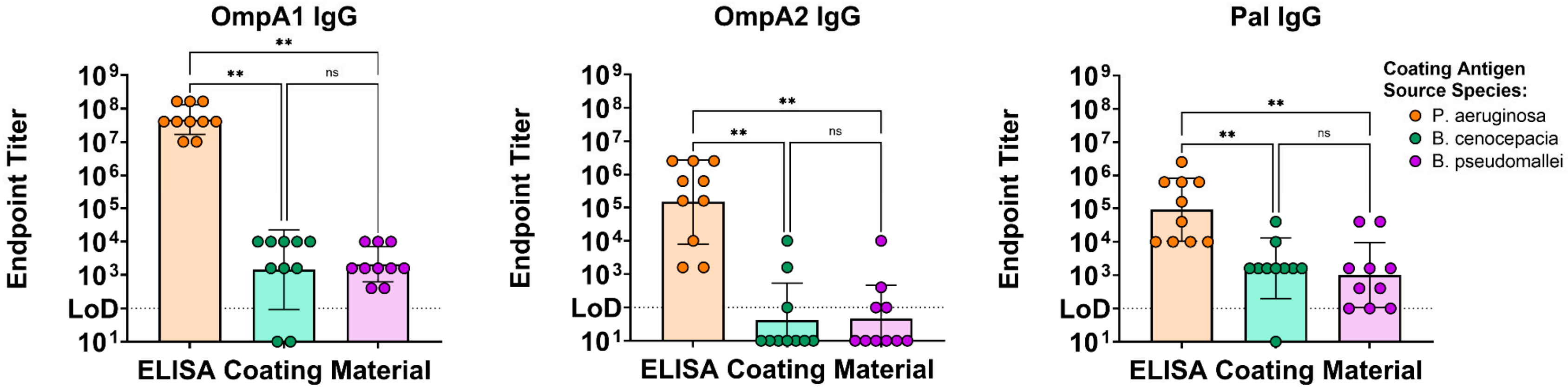

Figure S3: ELISAs using serum from mice immunized with (**a**) PaOmpA1, (**b**) PaOmpA2, or (**c**) PaPal. For each vaccination group, three separate ELISAs were performed which differed only in the protein that was coated on the ELISA plates. The PaOmpA1 group serum was probed against BpmOmpA1, Bcc OmpA1, and PaOmpA1; the PaOmpA2 group serum was probed against BpmOmpA2, BccOmpA2, and PaOmpA2; and the PaPal group serum was probed against BpmPal, BccPal, and PaPal. Endpoint titers correspond to the highest dilution at which signal from the immunization serum was >3 SD of the mean signal intensity of the equivalently diluted saline control serum. The limit of detection was the highest dilution evaluated, a factor of one hundred. Endpoint titers were compared via Friedman test with Dunn's post hoc tests. (**) $p < 0.01$.

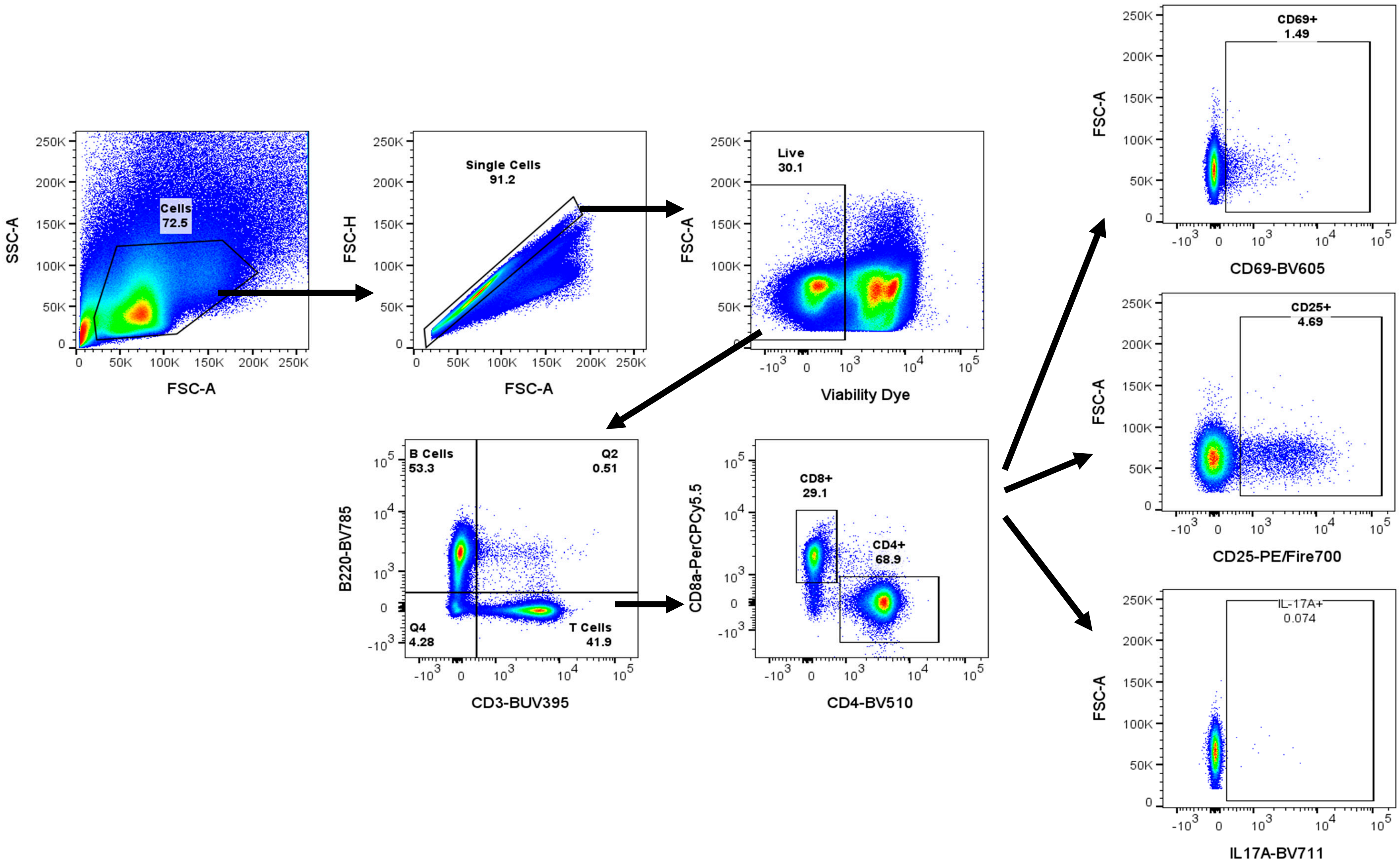

Figure S4: Gating strategy employed to analyze the flow cytometry data from the splenocyte antigen recall experiment.

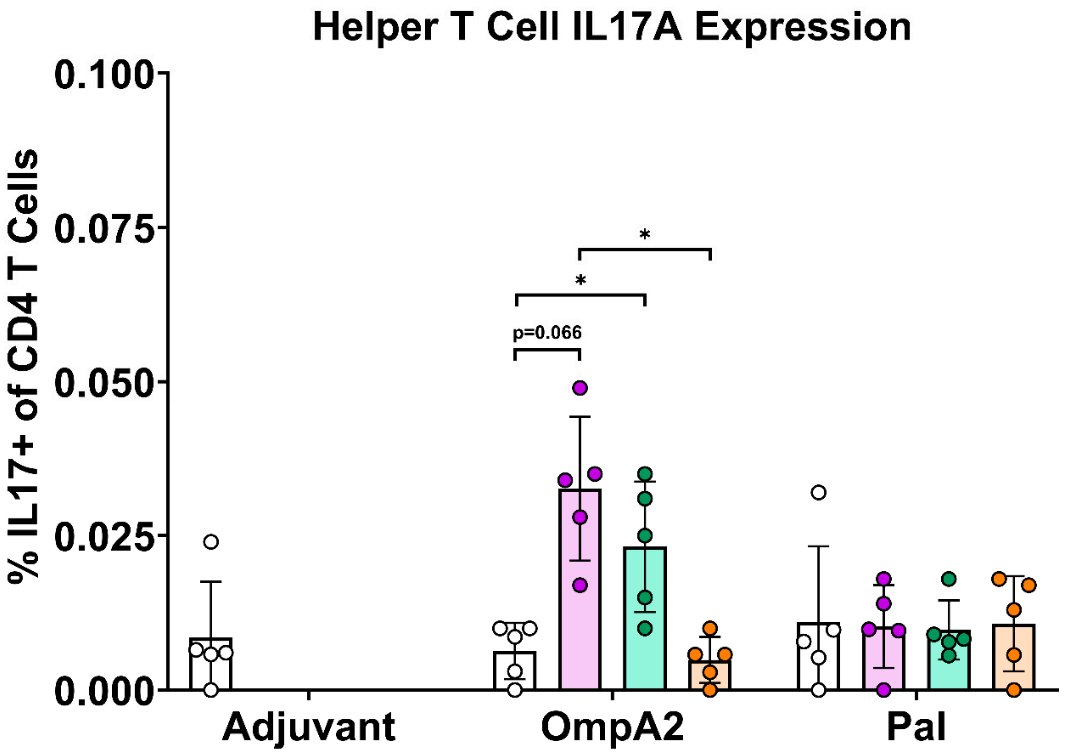

Figure S5: Intracellular IL17A fluorescent staining of antigen recalled splenocytes. Gated on CD3+B220-CD4+IL17A+ cells. The proportions of IL17A+ CD4 T cells were compared via two-way repeated measures ANOVA with Tukey post hoc tests. (*) $p < 0.05$.

Table S1: Primers and *E. coli* codon optimized gBlocks used for cloning. Purple corresponds to DNA overhangs. Yellow corresponds to *Nde*I/*Xho*I restriction sites. Green corresponds to the 6X-histidine tag. Codon optimization was performed with Integrated DNA Technologies' Codon Optimization Tool.

Primer/gBlock	Sequence
BccPal F Primer	tagtatcatatgcatcatcatcatcatcatAAGTCGGGCGTGAAGCTC
BccPal R Primer	tagtatctcgagTTACTGTTGGTAGACGAGGTC
BccOmpA1 gBlock	TAGTATCATATGCATCATCATCATCATCATGCCACGCAGCAGGGGAATAACACTGCGGTTGGGACTGGTACAGGAGCAGCATTAGGTGCT<br>GGCATCGGAGCTTTGGCAGGGGGCGGTAAAGGGGCGGCAATAGGAGCTGGCGTTGGTGCCTTAGTTGGCGGCGTTACGGGATATAACT<br>GGCAGGCGATAAAAAACAAGCTGGCACCTTCCGCCGCCCAGACAGGCACACAAGTTACGGAGCAACCGGATGGAAGTTTAAAGCTGA<br>ACGTACCTTCCAGTGTGACTTTTGCTACGAATCAATACGCAATAACACCGGCATTCACGCCGTTGTTAAACGATTTGGCTACAACACTGA<br>ACCAGAACCCGCAGGTGACCGCGTCCATTGTAGGTTATACTGACTCGACAGGATCAGCTCAGTTAAACCAGACGTTGAGTCAAAATAG<br>AGCGCAGTCCGTAGTTAACGCACTTGTCCAGCGTGGAGTTAATGGTGGTCGGTTGTCCGCCCAAGGTATGGGAGCGTCTAACCCAATTG<br>CGGACAATGCAACCGAAGCCGGTCGCGCCCAGAACAGAAGAGTTGAGATATATTTAAGAGCTCCGCAACAGCACCAGTAACTCGAGT<br>AGTAT
BccOmpA2 gBlock	TAGTATCATATGCATCATCATCATCATCATGTCGCACCTGCCATTACCTCGCAAAAGATAACTTACCAGGCTGATGCCTTGTTCGACTTCG<br>ATAAGGCTACACTGAAGCCCCTGGGTAAACAAAAACTGGACGAGTTGGCTTCAAAAATTGAGGGTATGAATACCGAAGTAGTAGTGGC<br>CACTGGTTACACCGACCGTATTGGGTCTGACAAATACAATGACCGCTTATCACTGCGTCGCGCCCAGGCGGTCAAGAGCTACTTAGTTA<br>GCAAGGGTGTCCCCGCTAACAAGATTTATACGGAGGGGAAAGGCAAACGTAACCCTGTTACGACTGGATGCAATCAAAAAAACAGAA<br>AACAACTTATAGCTTGTTTGGCACCGGACCGGCGGGTCGAAGTTGAAGTCGTGGGTACACAACAAGTGCAGAAAACAACAGTTCCGGC<br>GAACTAACTCGAGTAGTAT
PaOmpA1 gBlock	TAGTATCATATGCATCATCATCATCATCATGCCAATAACAATCCTTACGACACGCAGTCCCAGAGCCAGGGGGGATGTCAAAAACCGC<br>AAAATACGGCGGGTTGGGTGCACTTGCGGGAGCGGTAGCGGGTGCGGCGATTGATCACAACAACCGGGGAAAAGGGGCTCTTATAGG<br>CGCTGCTGTCGCGGGGGCCGCAGCAGCAGGTTATGGATATTATGCCGATAAGCAGGAAGCAGAACTTAGAAGACAAATGGAAGGTACA<br>GGGGTTGAAGTGCAGAGACAAGGGGATGACATAAAGTTAATAATGCCTGGGAATATCACCTTCGCCACTGATAGTGCAAATATTGCTCC<br>CTCCTTCTATGCGCCGCTGAACAATTTAGCCAACAGCTTTAAGCAGTATAACCAAAACACTATAGAGATAGTAGGCTATACTGATTCCAC<br>GGGCTCCCGTCAACATAACATGGACTTGTCTCAGCGGCGTGCACAGTCGGTTGCAGGGTATTTAACGGCGCAGGGTGTCGACGGTACAC<br>GGTTATCGACTCGGGGTATGGGTCCAGATCAACCGATTGCTTCTAATTCTACGGCTGACGGACGTGCCCAGAACCGCCGCGTTGAGGTG<br>AATTTGCGCCCTGTTCCCGGAGCACAAGGGCCTGCTCAAACTCAGCCGCAGTATTGACTCGAGTAGTAT
PaOmpA2 gBlock	TAGTATCATATGCATCATCATCATCATCATAAGCAGACTTCTCGCGGCACCATGGTGACGTTCGGAGATGTCCTTTTCGATCTGGACAAGA<br>GTGATCTTAAGCCAGGAGCAATGAGAAACATACAACAGCTTGCCGAGTTTCTGCAACAAAACCCGGAACGTCAGGTCATCGTTGAGGG<br>GTACACAGACAGTACAGGCTCCGCAAACTACAATCAGCGGTTAAGCGAACGCCGGGCAGACTCAGTTCGGATGGCGTTATTGAGTCGC<br>GGTATTTCGCCTGAGCGTGTCGCAACGCGCGGGTATGGTAAAGAGTATCCAGTGGCAAGCAACGGTACGTCGTCCGGCCGGGCTATGAA<br>CCGTCGGGTTGAGGTCACAATTAGCAACGATGCCAAACCTGTGGCCCCCCGCTCCAGTGTAAGCGGGTGACTCGAGTAGTAT
PaPal gBlock	TAGTATCATATGCATCATCATCATCATCATTCCAGCAAGGGTGGTGACGCTAGCGGGGAGGGGGCTAATGGCGGGGTTGACCCCAACGC<br>CGGGTATGGCGCCAATAGTGGCGCAGTAGATGGTAGTTTGTCAGACGAAGCTGCCCTTCGCGCGATAACCACTTTTTATTTCGAGTACGA<br>TTCATCCGACTTGAAGCCTGAGGCCATGCGGGCACTTGATGTCCACGCCAAAGACTTAAAAGGCTCCGGCCAACGGGTTGTTCTTGAAG<br>GGCACACTGATGAAAGAGGCACTCGTGAGTATAACATGGCATTGGGTGAACGGCGGGCTAAAGCAGTTCAGCGGTACCTTGTGTTACA<br>GGGAGTGTCGCCTGCACAACTTGAACTTGTCTCTTACGGGAAAGAACGGCCCGTGGCCACAGGCCACGACGAGCAATCCTGGGCACA<br>AAACCGCCGTGTGGAGTTGAAAAAGTAACTCGAGTAGTAT

Table S2: Corrected *p* values from Friedman tests with Dunn's *post hoc* used in Figure 3.

	BccOmpA1	PaOmpA1	BccOmpA2	PaOmpA2	BccPal	PaPal
**BpmOmpA1**	>0.9999 (ns)	0.0001 (***)				
**BpmOmpA2**			0.9304 (ns)	<0.0001 (****)		
**BpmPal**					>0.9999 (ns)	0.6306 (ns)

Table S3: Whole bacteria ELISA endpoint titers. The limit of detection (LoD) was the lowest dilution of serum evaluated, a factor of one hundred.

Immunization Group	Animal Number	*Bpm* Endpoint Titer	*Bm* Endpoint Titer	*Bmv* Endpoint Titer	*Bcc* Endpoint Titer	*P. aeruginosa* Endpoint Titer
OmpA1	1	1600	400	25600	Below LoD	Below LoD
	2	400	400	25600	Below LoD	Below LoD
	3	6400	6400	102400	Below LoD	Below LoD
	4	6400	6400	102400	Below LoD	Below LoD
	5	1600	1600	25600	6400	25600
	6	25600	25600	102400	Below LoD	Below LoD
	7	6400	6400	102400	Below LoD	Below LoD
	8	6400	6400	102400	Below LoD	Below LoD
	9	6400	1600	102400	Below LoD	Below LoD
	10	1600	6400	409600	Below LoD	Below LoD
	11	1600	6400	409600	Below LoD	Below LoD
	12	6400	6400	1638400	Below LoD	Below LoD
	13	6400	6400	102400	Below LoD	Below LoD
	14	400	400	25600	400	Below LoD
	15	6400	6400	409600	Below LoD	25600
OmpA2	1	25600	6400	102400	6400	Below LoD
	2	25600	6400	409600	102400	Below LoD
	3	6400	6400	409600	6400	400
	4	6400	25600	409600	6400	Below LoD
	5	6400	6400	25600	Below LoD	Below LoD
	6	1600	6400	6400	6400	Below LoD
	7	6400	6400	6400	6400	Below LoD
	8	6400	6400	25600	6400	Below LoD
	9	6400	6400	25600	Below LoD	Below LoD
	10	6400	6400	25600	Below LoD	Below LoD
	11	6400	6400	6400	400	102400
	12	25600	25600	25600	6400	102400
	13	6400	6400	25600	102400	409600
	14	6400	6400	25600	6400	102400
	15	400	400	102400	6400	409600
Pal	1	400	100	6400	Below LoD	Below LoD
	2	Below LoD	100	Below LoD	Below LoD	Below LoD
	3	1600	1600	6400	409600	102400
	4	400	100	Below LoD	Below LoD	Below LoD
	5	Below LoD	Below LoD	Below LoD	Below LoD	Below LoD
	6	Below LoD	Below LoD	Below LoD	Below LoD	Below LoD
	7	Below LoD	100	1600	Below LoD	Below LoD
	8	100	100	6400	Below LoD	Below LoD
	9	400	100	25600	Below LoD	Below LoD
	10	Below LoD	Below LoD	6400	Below LoD	Below LoD
	11	400	1600	6400	102400	102400
	12	Below LoD	Below LoD	6400	Below LoD	Below LoD
	13	400	100	6400	6400	409600
	14	400	400	6400	Below LoD	25600
	15	100	100	25600	1600	25600

Table S4: Corrected *p* values from Kruskal-Wallis tests with Dunn's *post hoc* used in Figure 4.

ELISA COATING SPECIES		BpmOmpA1	BpmOmpA2	BpmPal
***Bpm* Bp82**	**BpmOmpA1**		0.7863 (ns)	0.7863 (ns)
	**BpmOmpA2**	0.7863 (ns)		<0.0001 (****)
	**BpmPal**	0.7863 (ns)	<0.0001 (****)	
***Bm* CLH001**	**BpmOmpA1**		0.8865 (ns)	<0.0001 (****)
	**BpmOmpA2**	0.8865 (ns)		<0.0001 (****)
	**BpmPal**	<0.0001 (****)	<0.0001 (****)	
***Bmv* ATCC 17616**	**BpmOmpA1**		0.3215 (ns)	<0.0001 (****)
	**BpmOmpA2**	0.3215 (ns)		0.0021 (**)
	**BpmPal**	<0.0001 (****)	0.0021 (**)	
***Bcc* K56-2**	**BpmOmpA1**		0.0006 (***)	0.9892 (ns)
	**BpmOmpA2**	0.0006 (***)		0.0188 (*)
	**BpmPal**	0.9892 (ns)	0.0188 (*)	
***Pa* PA103**	**BpmOmpA1**		0.2230 (ns)	0.5574 (ns)
	**BpmOmpA2**	0.2230 (ns)		>0.9999 (ns)
	**BpmPal**	0.5574 (ns)	>0.9999 (ns)	

Table S5: Corrected *p* values from two-way repeated measures ANOVAs with Tukey's *post hoc* used in Figure 5.

		BpmOmpA2 Vaccination Group				BpmPal Vaccination Group			
		**Unstimulated**	**BpmOmpA2**	**BccOmpA2**	**PaOmpA2**	**Unstimulated**	**BpmPal**	**BccPal**	**PaPal**
**CD4+CD25+ (Fig 5a)**	**Unstimulated**		0.0238 (*)	0.0147 (*)	0.8331 (ns)		0.0162 (*)	0.0047 (**)	0.7030 (ns)
	**Bpm-(OmpA2/Pal)**	0.0238 (*)		0.9548 (ns)	0.0735 (ns)	0.0162 (*)		0.0342 (*)	0.0931 (ns)
	**Bcc-(OmpA2/Pal)**	0.0147 (*)	0.9548 (ns)		0.0441 (*)	0.0047 (**)	0.0342 (*)		0.0087 (**)
	**Pa-(OmpA2/Pal)**	0.8331 (ns)	0.0735 (ns)	0.0441 (*)		0.7030 (ns)	0.0931 (ns)	0.0087 (**)	
**CD4+CD69+ (Fig 5b)**	**Unstimulated**		0.0224 (*)	0.0005 (***)	0.5247 (ns)		0.0201 (*)	0.0043 (**)	0.0590 (ns)
	**Bpm-(OmpA2/Pal)**	0.0224 (*)		0.8309 (ns)	0.0969 (ns)	0.0201 (*)		0.0084 (**)	0.0021 (**)
	**Bcc-(OmpA2/Pal)**	0.0005 (***)	0.8309 (ns)		0.0123 (*)	0.0043 (**)	0.0084 (**)		0.0031 (**)
	**Pa-(OmpA2/Pal)**	0.5247 (ns)	0.0969 (ns)	0.0123 (*)		0.0590 (ns)	0.0021 (**)	0.0031 (**)	
**CD8+CD25+ (Fig 5c)**	**Unstimulated**		0.1066 (ns)	0.1311 (ns)	0.8697 (ns)		0.0410 (*)	0.0178 (*)	0.6495 (ns)
	**Bpm-(OmpA2/Pal)**	0.1066 (ns)		0.3784 (ns)	0.0989 (ns)	0.0410 (*)		0.0509 (ns)	0.0134 (*)
	**Bcc-(OmpA2/Pal)**	0.1311 (ns)	0.3784 (ns)		0.1236 (ns)	0.0178 (*)	0.0509 (ns)		0.0078 (**)
	**Pa-(OmpA2/Pal)**	0.8697 (ns)	0.0989 (ns)	0.1236 (ns)		0.6495 (ns)	0.0134 (*)	0.0078 (**)	
**CD8+CD69+ (Fig 5d)**	**Unstimulated**		0.0662 (ns)	0.0310 (*)	0.9496 (ns)		0.0083 (**)	0.0500 (ns)	0.8189 (ns)
	**Bpm-(OmpA2/Pal)**	0.0662 (ns)		0.0050 (**)	0.0943 (ns)	0.0083 (**)		0.1039 (ns)	0.0262 (*)
	**Bcc-(OmpA2/Pal)**	0.0310 (*)	0.0050 (**)		0.0437 (*)	0.0500 (ns)	0.1039 (ns)		0.0431 (*)
	**Pa-(OmpA2/Pal)**	0.9496 (ns)	0.0943 (ns)	0.0437 (*)		0.8189 (ns)	0.0262 (*)	0.0431 (*)	

Table S6: Corrected *p* values from two-way repeated measures ANOVAs with Šidák's *post hoc* used in Figure 6.

		Adj Unstimulated	Adj BpmOmpA2	Adj BccOmpA2	Adj PaOmpA2	Vax Unstimulated	Vax BpmOmpA2	Vax BccOmpA2	Vax PaOmpA2
**IFNγ ELISPOT**	**Adj Unstimulated**		Undefined	Undefined	Undefined	Undefined			
	**Adj BpmOmpA2**	Undefined		Undefined	Undefined		0.0496 (*)		
	**Adj BccOmpA2**	Undefined	Undefined		Undefined			0.0134 (*)	
	**Adj PaOmpA2**	Undefined	Undefined	Undefined					0.1419 (ns)
	**Vax Unstimulated**	Undefined					0.1518 (ns)	0.0443 (*)	0.3803 (ns)
	**Vax BpmOmpA2**		0.0496 (*)			0.1518 (ns)		0.9920 (ns)	0.1469 (ns)
	**Vax BccOmpA2**			0.0134 (*)		0.0443 (*)	0.9920 (ns)		0.0390 (*)
	**Vax PaOmpA2**				0.1419 (ns)	0.1419 (ns)	0.1469 (ns)	0.0390 (*)	
**IL-17A ELISPOT**	**Adj Unstimulated**		Undefined	Undefined	0.7586 (ns)	Undefined			
	**Adj BpmOmpA2**	Undefined		Undefined	0.7586 (ns)		0.0888 (ns)		
	**Adj BccOmpA2**	Undefined	Undefined		0.7586 (ns)			0.0052 (**)	
	**Adj PaOmpA2**	0.7586 (ns)	0.7586 (ns)	0.7586 (ns)					0.6707 (ns)
	**Vax Unstimulated**	Undefined					0.2558 (ns)	0.0176 (*)	0.7586 (ns)
	**Vax BpmOmpA2**		0.0888 (ns)			0.2558 (ns)		0.8240 (ns)	0.2485 (ns)
	**Vax BccOmpA2**			0.0052 (**)		0.0176 (*)	0.8240 (ns)		0.0143 (*)
	**Vax PaOmpA2**				0.6707 (ns)	0.7586 (ns)	0.2485 (ns)	0.0143 (*)	
